# *Arap1* loss causes retinal pigment epithelium phagocytic dysfunction and subsequent photoreceptor death

**DOI:** 10.1242/dmm.049343

**Published:** 2022-07-25

**Authors:** Andy Shao, Antonio Jacobo Lopez, JiaJia Chen, Addy Tham, Seanne Javier, Alejandra Quiroz, Sonia Frick, Edward M. Levine, K. C. Kent Lloyd, Brian C. Leonard, Christopher J. Murphy, Thomas M. Glaser, Ala Moshiri

**Affiliations:** 1 The University of Nevada, Reno School of Medicine, Reno, NV 89557, USA; 2 Department of Ophthalmology and Vision Science, School of Medicine, UC Davis, Sacramento, CA 95817, USA; 3 Department of Ophthalmology and Visual Sciences, Vanderbilt University, Nashville, TN 37235, USA; 4 Mouse Biology Program, UC Davis, Davis, CA 95616, USA; 5 Department of Surgery, School of Medicine, UC Davis, Sacramento, CA 95817, USA; 6 Department of Surgical and Radiological Sciences, School of Veterinary Medicine, UC Davis, Davis, CA 95616, USA; 7 Department of Cell Biology and Human Anatomy, School of Medicine, UC Davis, Davis, CA 95616, USA

**Keywords:** *Arap1*, RPE, Phagocytosis, Retinitis, Pigmentosa

## Abstract

Retinitis pigmentosa (RP), a retinal degenerative disease, is the leading cause of heritable blindness. Previously, we described that *Arap1^−/−^* mice develop a similar pattern of photoreceptor degeneration. *Arap1* is an Arf-directed GTPase-activating protein shown to modulate actin cytoskeletal dynamics. Curiously, *Arap1* expression was detected in Müller glia and retinal pigment epithelium (RPE), but not the photoreceptors themselves. In this study, we generated conditional knockout mice for Müller glia/RPE, Müller glia and RPE via targeting *Rlbp1*, *Glast* and *Vmd2* promoters, respectively, to drive Cre recombinase expression to knock out *Arap1*. *Vmd2-Cre Arap1^tm1c/tm1c^* and *Rlbp1-Cre Arap1^tm1c/tm1c^* mice, but not *Glast-Cre Arap1^tm1c/tm1c^* mice, recapitulated the phenotype originally observed in germline *Arap1^−/−^* mice. Mass spectrometry analysis of human ARAP1 co-immunoprecipitation identified candidate binding partners of ARAP1, revealing potential interactants involved in phagocytosis, cytoskeletal composition, intracellular trafficking and endocytosis. Quantification of outer segment phagocytosis *in vivo* demonstrated a clear phagocytic defect in *Arap1^−/−^* mice compared to *Arap1^+/+^* controls. We conclude that *Arap1* expression in RPE is necessary for photoreceptor survival due to its indispensable function in RPE phagocytosis.

This article has an associated First Person interview with the first author of the paper.

## Abstract (precis)

**Summary:** We provide evidence that *Arap1* expression in retinal pigment epithelium (RPE) is essential for maintaining photoreceptor health due to its indispensable role in RPE phagocytosis.

## INTRODUCTION

Retinitis pigmentosa (RP) is a rod/cone dystrophy characterized by progressive photoreceptor loss accompanied by striking pigmentary changes on fundus examination (Hamel, 2006). Generally, cell death follows a progression of early rod loss followed by cone degeneration (Hamel, 2006). Currently, RP is the leading cause of heritable blindness, with over 80 gene mutations, identified for its nonsyndromic form (Verbakel et al., 2018).

Previously, we discovered that *Arap1^−/−^* mice, generated by the National Institutes of Health Knockout Mouse Phenotyping Program (KOMP2), develop a phenotype similar to human RP (Moshiri et al., 2017). *Arap1* is an Arf-directed GTPase-activating protein (GAP) with a RhoGAP and multiple pleckstrin homology (PH) domains. As such, *Arap1* governs a diverse range of functions. *Arap1* has been shown to facilitate EGFR endocytosis and consequent regulation of EGFR signal transduction (Yoon et al., 2008). Additionally, *Arap1* has been shown to play a key role in actin modulation as an intersecting node for both Arf- and Rho-directed actin dynamics (Miura et al., 2002). However, *Arap1* function in the retina had not yet been assessed prior to our investigations.

*Arap1^−/−^* mice demonstrated optic nerve pallor, attenuated retinal arteries, retinal pigmentary changes and focal areas of retinal pigment epithelium (RPE) atrophy, a constellation of findings consistent with those seen in human RP. Optical coherence tomography (OCT) and histopathology revealed outer retinal degradation, most marked in the outer nuclear layer (ONL), with inner retina preservation. These changes were corroborated by reduced scotopic responses and later photopic signal reduction in electroretinography (ERG). As we investigated mechanistic explanations for this phenotype, we found that *Arap1^−/−^* mice experienced normal retinal histogenesis, although soon followed by progressive photoreceptor loss. Despite the photoreceptor degeneration observed in the knockout, *Arap1* was not found to be expressed in the photoreceptors, but rather in the adjacent support cells: Müller glia and RPE. In our initial study (Moshiri et al., 2017), *Arap1* expression was difficult to assess in the RPE due to its pigmented nature.

Müller glia are retinal cells that govern multiple essential functions in retinal homeostasis, including maintenance of retinal structural integrity, glucose metabolism and neurotransmitter reuptake (Reichenbach and Bringmann, 2013). The RPE is a monolayer of cells located beneath the photoreceptor layer known to manage the transport of nutrients, ions and water, protect against photooxidation, re-isomerize all-trans-retinal into 11-cis-retinal, secrete essential factors for retinal maintenance and phagocytose photoreceptor outer segments (OSs) (Simó et al., 2010). Dysfunction in both cell types has been implicated in the development and pathology of RP. For instance, *MERTK*, encoding a receptor tyrosine kinase involved in RPE phagocytosis, and *RPE65*, encoding an isomerohydrolase essential for visual pigment recycling, have both been linked as RP-causative mutations in the RPE (Ferrari et al., 2011; Gal et al., 2000). Reactive gliosis governed by Müller glia has been described extensively in RP models, although not many single gene disorders of the retina are directly linked to Müller glial-specific genes (Hippert et al., 2015). To date, *Arap1* has not been established to play a role in photoreceptor survival in either of these two cell types. Our current investigations seek to elucidate the mechanistic link between *Arap1* loss in Müller glia and/or RPE cells and subsequent development of an RP-like phenotype in mice.

## RESULTS

### Loss of *Arap1*, expressed in Müller glia and RPE, is associated with photoreceptor death

**Fig. 1.**
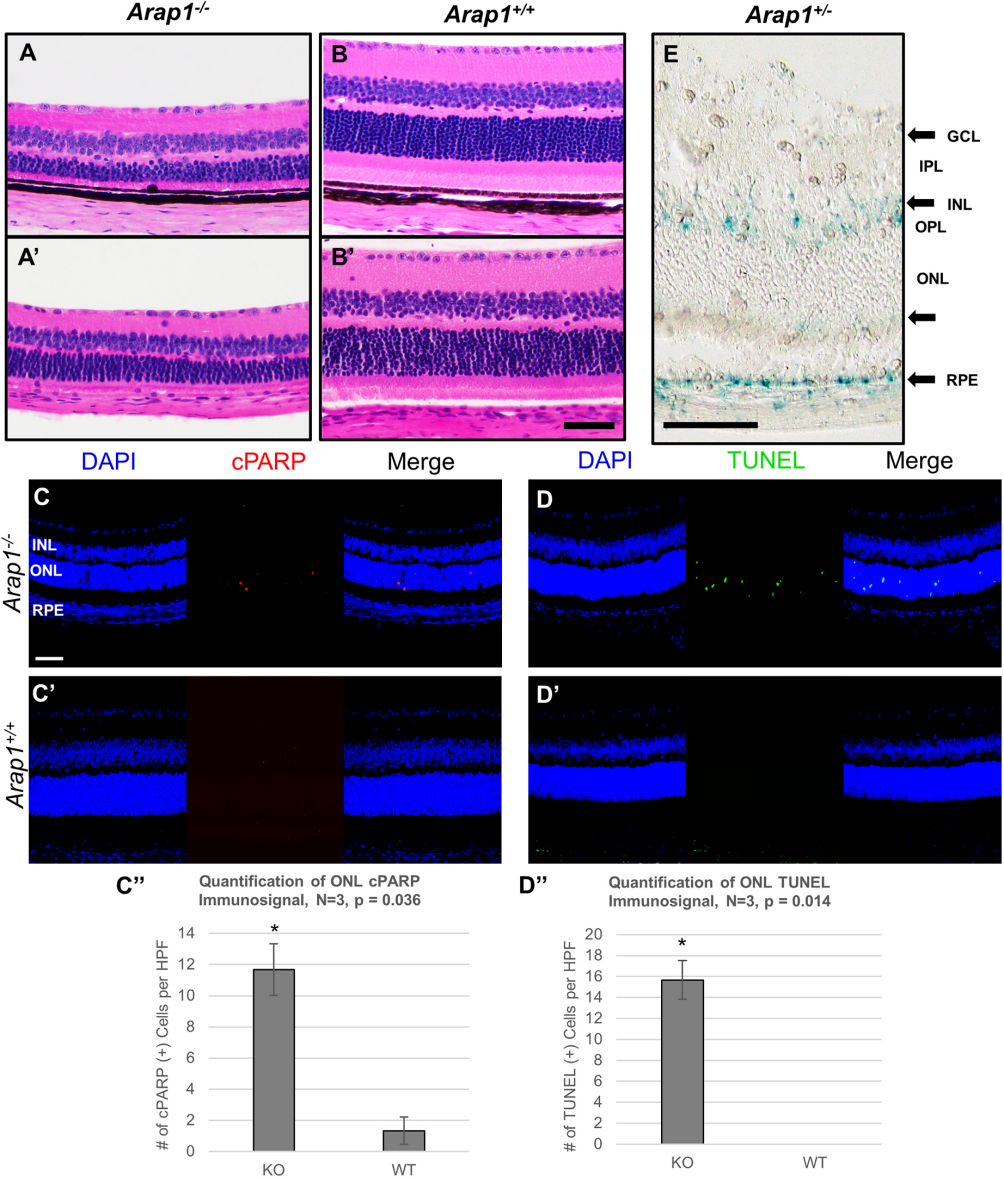
**Characterization of albino *Arap1*^−/−^ retina.** (A-B′) Haematoxlin and Eosin (H&E) staining of representative retinal sections of pigmented and albino *Arap1^−/−^* (A,A′) and *Arap1^+/+^* (B,B′) mice. Quantifications of retinal layers in albino and pigmented wild-type (WT) and mutant animals are shown in Fig. S1. Outer retinal dysplasia and photoreceptor degeneration are prominent in both pigmented and albino *Arap1^−/−^* retinas but not seen in *Arap1^+/+^* retinas. (C,C′,D,D′) Representative retinal sections of albino *Arap1^−/−^* (C,D) and *Arap1^+/+^* (C′,D′) mice were used for cleaved PARP-1 (cPARP, red; C,C′) fluorescent staining immunohistochemistry and terminal deoxynucleotidyl transferase dUTP nick end labeling assay (TUNEL, green; D,D′) to assess programmed cell death. DAPI counterstaining (blue) was used to visualize the nuclei of the retinal layers. Both cPARP and TUNEL signals were prominent in the outer nuclear layer (ONL) of *Arap1^−/−^* retinas but minimal to nonexistent in control *Arap1^+/+^* retinas. (C″,D″) Quantification of cPARP and TUNEL immunosignal is shown for both *Arap1^−/−^* (KO) and WT animals per 40× high-power field (HPF). (E) Retinal sections of *Arap1^+/−^* mice were stained with X-gal to assess LacZ histochemical reaction. Blue X-gal signal was detected in the inner nuclear layer (INL) and retinal pigment epithelium (RPE), with weaker signal in the outermost aspect of the ONL and ganglion cell layer (GCL; arrows). cPARP, TUNEL and X-gal analysis were performed on tissue harvested from mice aged P24; H&E staining was performed on tissue harvested from mice aged 6 weeks postnatal. The GCL, inner plexiform layer (IPL), INL, outer plexiform layer (OPL), ONL, inner segment (IS), outer segment (OS) and RPE are labeled in E. All images were taken at 40× magnification. Scale bars: 100 μm. *n*≥3 for each group, tissue was collected from three different animals of each respective genotype, average values represent the mean, error bars represent s.e.m. Significance calculated by two-tailed, unpaired Student's *t*-test, *P<0.05.

Previously, we reported photoreceptor degeneration in *Arap1^−/−^* mice on a pigmented C57BL/6J background (Moshiri et al., 2017). Using the LacZ cassette knocked into the *Arap1* locus under control of its promoter, we were able to use an X-gal reaction to detect *Arap1* expression in the retina. However, pigment in the RPE layer made X-gal signal detection and subsequent verification of *Arap1* expression difficult. To better assess RPE *Arap1* expression, we bred *Arap1^−/−^* mice onto a *Tyr^−/−^* C57BL/6J albino background. To confirm that the pattern of retinal degeneration seen originally in the pigmented *Arap1^−/−^* mice was still present, we sectioned eyes and stained with Hematoxylin and Eosin (H&E) to visualize retinal morphology. Albino *Arap1^−/−^* mice exhibited thinning of the ONL and preservation of inner retinal morphology (Fig. 1A′), identical to pigmented *Arap1^−/−^* mice (Fig. 1A). These changes were absent in albino and pigmented *Arap1^+/+^* littermates (Fig. 1B,B′). Quantification of these observations revealed a statistically significant reduction in the ONL, inner segment (IS) and OS layers of mutant animals compared to wild-type (WT) littermates in both pigmented and albino mice (Fig. S1).

To assess whether photoreceptor loss was due to programmed cell death, we analyzed apoptotic signal with both terminal deoxynucleotidyl transferase dUTP nick end labeling (TUNEL) and cleaved PARP-1 (cPARP) fluorescent staining. TUNEL staining visualizes DNA fragmentation secondary to apoptosis, while cPARP staining measures caspase-3 activity. Consistent with the pattern of degeneration observed on H&E staining, apoptotic signals were observed in the ONL of *Arap1^−/−^* mice in both TUNEL and cPARP fluorescence (Fig. 1C,D). In contrast, apoptotic signals were absent to near absent in the ONL in *Arap1^+/+^* control retinas (Fig. 1C′,D′). Immunosignal for both assays was quantified (Fig. 1C″,D″).

To assess *Arap1* expression in the RPE, we used our albino *Arap1^+/−^* mice and the signal from the X-gal histochemical reaction as a surrogate for *Arap1* expression. In postnatal day (P)24 retina, blue X-gal signal was clearly seen in the RPE and the inner nuclear layer (INL) of albino *Arap1^+/−^* mice, with faint signal in the ganglion cell layer (GCL) and outermost aspect of the ONL (Fig. 1E). We confirmed previously that the expression pattern observed in these layers was due to Müller glia (Moshiri et al., 2017). However, signal was also observed in the RPE layer, which was originally difficult to visualize in pigmented *Arap1^+/−^* mice, and clearly visible on an albino background (Fig. 1E).

### Conditional knockout (cKO) of *Arap1* in RPE, but not Müller glia, recapitulates the *Arap1^−/−^* photoreceptor degeneration phenotype

**Fig. 2.**
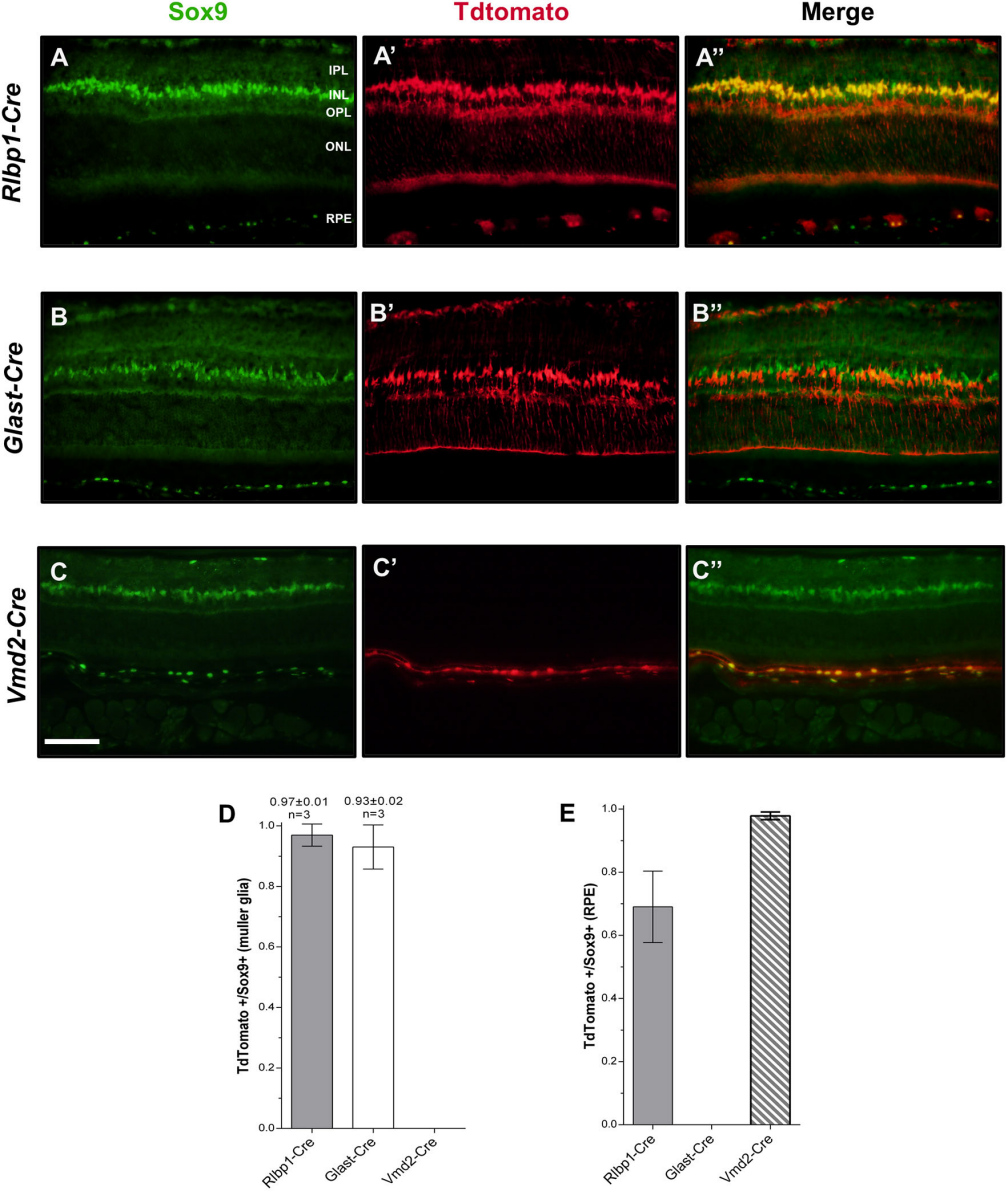
**Quantification of Cre function in conditional knockout (cKO) mice.** (A-C″) Immunohistochemistry was performed using anti-Sox9 (green) and anti-TdTomato (red) to quantify Cre function in *Rlbp1-Cre*, *Glast-Cre* and *Vmd2-Cre* mice at P84. (A,B,C) Sox9 immunosignal was detected in all of the Müller glia and RPE cells in all *Cre* lines. (A′,B′,C′) TdTomato signal was visualized in *Rlbp1-Cre*, *Glast-Cre* and *Vmd2-Cre* mouse lines. Channels were merged to create a composite image. TdTomato signal was present in the Müller glia in the *Rlbp1-Cre* and *Glast-Cre* strains, but not the *Vmd2-Cre* strain. Conversely, TdTomato signal was present in RPE in the *Rlbp1-Cre* and *Vmd2-Cre* strains, but not the *Glast-Cre* strain. (A″,B″,C″) Merged TdTomato/Sox9 images are shown. (D,E) Graphical quantification of the proportion of Sox9-positive cells that were also TdTomato positive in Müller glia (D) and RPE (E) for each *Cre* line. The IPL, INL, OPL, ONL and RPE are labeled in A. Images were taken at 40× magnification. Scale bar: 100 μm. *n*=3 for each group, tissue collected from three different animals of each respective genotype, error bars represent s.e.m.

To confirm that Arap1 expression specifically in the retina is essential for photoreceptor viability, particularly in Müller glia and RPE as X-gal reactivity suggested, we generated a tamoxifen-induced Müller glia/RPE-specific conditional *Arap1* knockout mouse. By using the *Rlbp1* promoter to drive *Cre* recombinase expression, knockout was ensured to be specific to Müller glia and RPE (Vogel et al., 2007). To assess both efficacy and specificity of targeting, Cre function in *Rlbp1-Cre* mice was quantified by breeding these mice onto an Ai9 background to generate a TdTomato signal (red) in cells with Cre activity. Anti-Sox9 (green), a transcription factor present in Müller glia and the RPE, was used to mark these nuclei for quantification of Cre function (Masuda et al., 2014; Poché et al., 2008). TdTomato fluorescence mirrored Sox9 immunosignal, confirming Cre function to be specific to the RPE and Müller glia (Fig. 2A,A′,A″). Cre function was present in nearly 100% of Müller glia and ∼70% of the RPE (Fig. 2D,E).

**Fig. 3.**
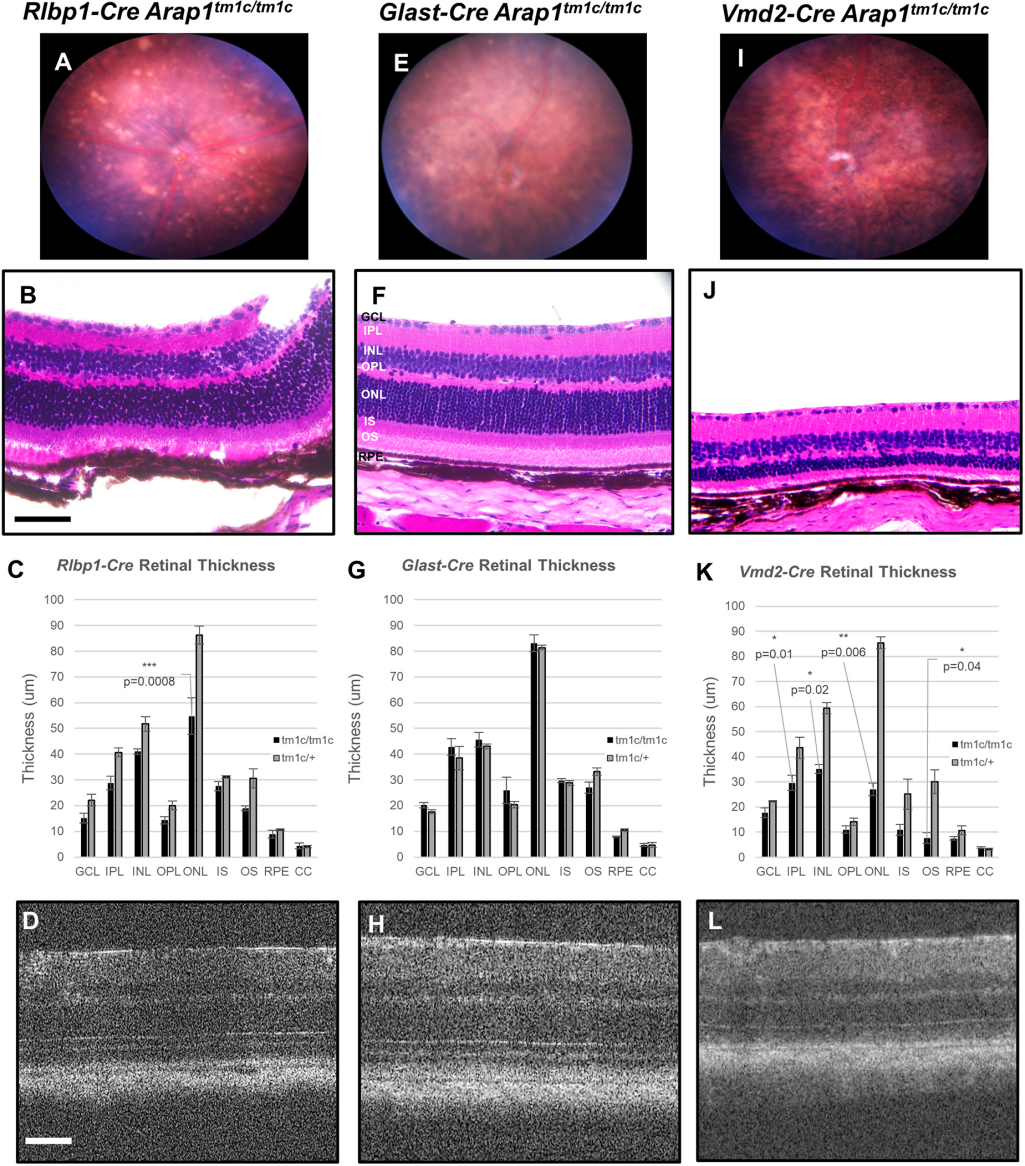
**Characterization of *Rlbp1-Cre Arap1^tm1c/tm1c^*, *Glast-Cre Arap1^tm1c/tm1c^* and *Vmd2-Cre Arap1^tm1c/tm1c^* cKO mouse lines.** *Cre* cKO mice were analyzed with fundus photography, histology and optical coherence tomography (OCT) at 3 months of age (*Glast-Cre*, *Rlbp1-Cre*) and 1 month of age (*Vmd2-Cre*). (A,I) Fundus photography demonstrated pigmentary changes, optic nerve pallor and vascular attenuation in the *Rlbp1-Cre Arap1^tm1c/tm1c^* and *Vmd2-Cre Arap1^tm1c/tm1c^* strains. (E) Conversely, the *Glast-Cre Arap1^tm1c/tm1c^* strain demonstrated no significant differences from WT littermates. (B,F,J) Representative retinal sections from the *Cre* strains were stained with H&E. (C,G,K) Quantification of retinal layers on histology is shown for each cKO line compared to conditional heterozygote controls. (B,C) *Rlbp1-Cre Arap1^tm1c/tm1c^* retinas demonstrated significant degeneration of the ONL with relative preservation of all other retinal layers. (F,G) *Glast-Cre Arap1^tm1c/tm1c^* retinas were indistinguishable from *Glast-Cre Arap1^tm1c/+^* littermate retinas. (J,K) *Vmd2-Cre Arap1^tm1c/tm1c^* retinas demonstrated more severe degeneration of the ONL (J), and quantification of retinal layers revealed significant degeneration of the IPL, INL and OS layers compared to those of heterozygous littermates (K). (D,H,L) These changes were consistent with *in vivo* OCT imaging of retinal layers. The GCL, IPL, INL, OPL, ONL, IS, OS and RPE are labeled in F. Scale bars: 100 μm. Images in B, F and J were taken at 40× magnification. *n*=3, tissue collected from three different animals of each respective genotype, average values represent the mean, error bars represent s.e.m. Significance calculated by two-tailed, unpaired Student's *t*-test, *P<0.05, **P<0.01, ***P<0.001.

*Rlbp1-Cre Arap1^tm1c/tm1c^* mice shared phenotypic features that were originally observed in *Arap1^−/−^* mice, although generally milder. Two months post-tamoxifen induction, fundus examination revealed retinal pigmentary changes, focal areas of RPE atrophy and areas of spotty hyper-reflective material (Fig. 3A). Histopathology revealed degenerative loss of the ONL as well as degenerative changes of the outer retina (Fig. 3B,C). These findings were corroborated by OCT imaging revealing thinning of the outer retina with preservation of the inner retina (Fig. 3D).

To ascertain whether the role of *Arap1* in Müller glia or RPE was determinant for the *Arap1^−/−^* phenotype, cKOs using *Glast* (also known as *Slc1a3*) and *Vmd2* (also known as *Best1*) promoters, respectively, were generated using the same method as for the *Rlbp1-Cre* mice. The *Vmd2* promoter is RPE specific, whereas the *Glast* promoter is Müller glia specific (Esumi et al., 2007; Slezak et al., 2007). Endogenous fluorescence of TdTomato and immunohistochemistry using anti-Sox9 was used again to quantify the Cre function in both the *Glast-Cre* and *Vmd2-Cre* lines (Fig. 2B,B′,C,C′). *Glast-Cre* TdTomato signal mirrored Sox9 signal in Müller glia, although lacked any observable signal in RPE (Fig. 2B″). *Vmd2-Cre* TdTomato signal, contrastingly, was only detected in the RPE layer (Fig. 2C″). Both knockouts expressed *Cre* in nearly 100% of their respective cells (Fig. 2D,E), confirming generation of Müller glia and RPE cell type-specific cKOs, respectively.

Despite the significant degree of *Arap1* expression in Müller glia, *Glast-Cre Arap1^tm1c/tm1c^* mice were phenotypically indistinguishable from WT mice. Fundus examination, histochemistry and OCT analysis revealed no significant changes from heterozygous littermates (Fig. 3E-H). In contrast, *Vmd2-Cre Arap1^tm1c/tm1c^* mice recapitulated the phenotype observed in *Rlbp1-Cre Arap1^tm1c/tm1c^* and *Arap1^−/−^* mice (Fig. 3I-L). Fundus examination again revealed optic nerve pallor, attenuated retinal arteries and retinal pigmentary changes, alongside outer retinal thinning on histology and OCT. However, histopathology was more severe than that observed in *Rlbp1-Cre Arap1^tm1c/tm1c^* mice, with significant degeneration of the inner plexiform layer (IPL), INL, ONL and OS compared to heterozygous littermates (Fig. 3J). Quantification of retinal layers per OCT analysis of both *Vmd2-Cre Arap1^tm1c/tm1c^* and *Vmd2-Cre Arap1^tm1c/+^* mice revealed similar degeneration in *Vmd2-Cre Arap1^tm1c/tm1c^* retinas, notably in the ONL and photoreceptor layers, with subsequent loss of total retinal thickness compared to that in heterozygous littermates (Fig. S2). This pattern of degeneration was similar to that previously observed in the original *Arap1*^−/−^ mice at similar chronological stages, with respect to induction (Moshiri et al., 2017).

Functional analysis of -*Cre Arap1^tm1c/tm1c^* and -*Cre Arap1^tm1c/+^* mice with ERG mirrored the degenerative patterns seen on histopathology and OCT. *Rlbp1-Cre Arap1^tm1c/tm1c^* mice demonstrated mildly diminished scotopic response, but intact photopic response (Fig. S3C). *Glast-Cre Arap1^tm1c/tm1c^* mice had responses indistinguishable from those of heterozygous littermates (Fig. S3B). *Vmd2-Cre Arap1^tm1c/tm1c^* mice had the most severe impairments of the three lines, with significant reduction in magnitude of the scotopic response and notable reduction in magnitude of photopic response (Fig. S3A).

H&E staining revealed invasion of the outer retina of *Vmd2-Cre Arap1^tm1c/tm1c^* and *Rlbp1-Cre Arap1^tm1c/tm1c^* retinas by cells that were suspected to be macrophages, absent in *Glast-Cre Arap1^tm1c/tm1c^* retinas (Fig. S4A-C). To confirm this finding, immunohistochemistry was performed using anti-CD11b to visualize retinal macrophages. Analysis revealed CD11b (also known as ITGAM) signal in the outer retina of *Rlbp1-Cre Arap1^tm1c/tm1c^* and *Vmd2-Cre Arap1^tm1c/tm1c^* mice, indicative of macrophage invasion (Fig. S4D″,F″). *Glast-Cre Arap1^tm1c/tm1c^* mice did not demonstrate any measurable CD11b signal in their retinas (Fig. S4E″).

**Fig. 4.**
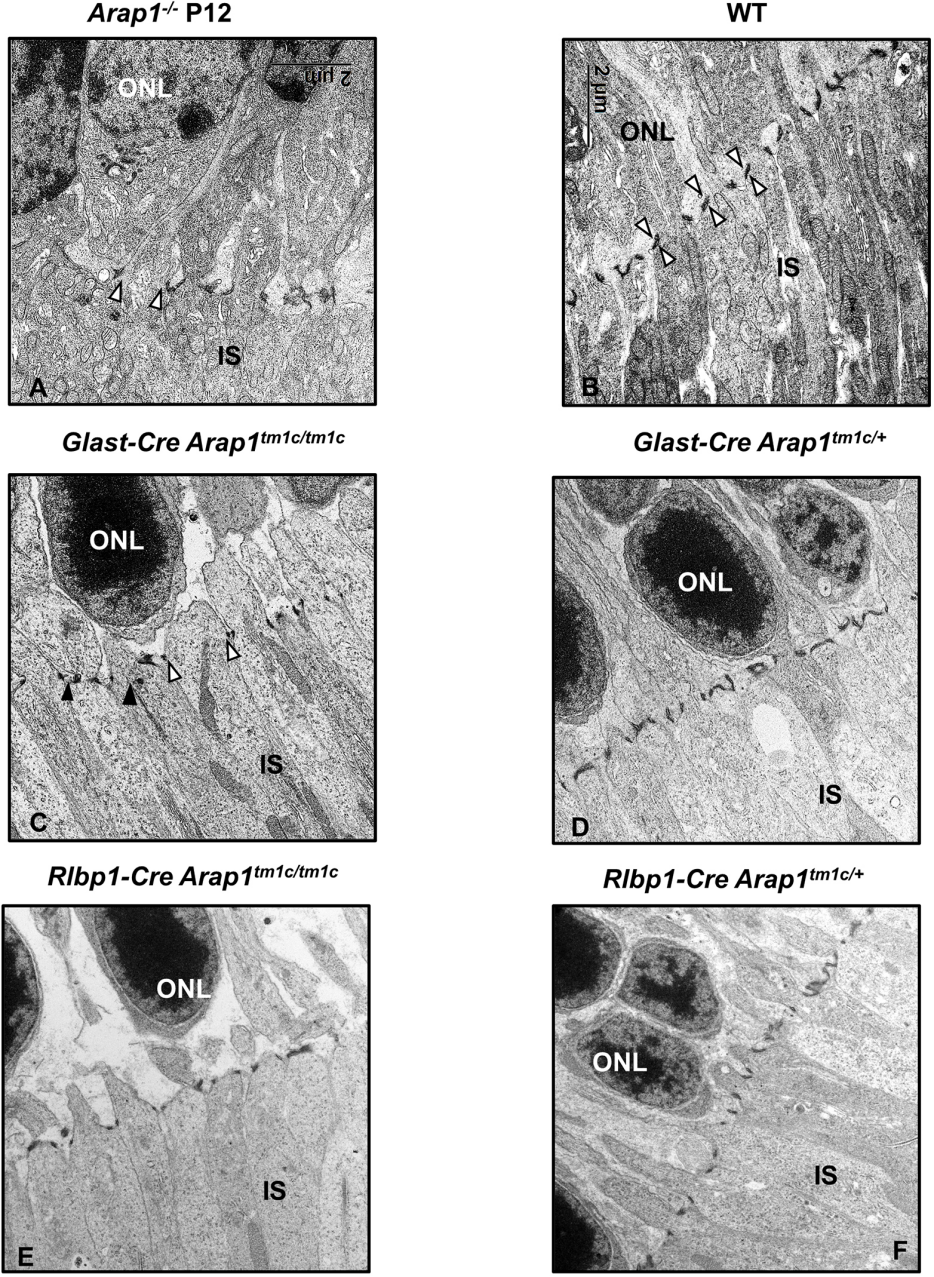
**External limiting membrane degeneration in germline and conditional *Arap1* knockout retinas.** Transmission electron microscopy of representative retinal sections in WT, *Arap1^−/−^*, *Glast-Cre Arap1^tm1c/tm1c^* and *Rlbp1-Cre Arap1^tm1c/tm1c^* mice. WT and *Arap1^−/−^* retinas were assessed at P12. *Glast*-*Cre Arap1^tm1c/tm1c^* and *Rlbp1*-*Cre Arap1^tm1c/+^* retinas were assessed at 8 months of age. (A) *Arap1^−/−^* retinas demonstrated fewer adherens junction (AJ) complexes with increased space between each junction (indicated by the white arrowheads) as well as loss of their linear morphology and arrangement. (B) WT retinas demonstrated normal architecture of AJ complexes (white arrowheads) with typical linear arrangement. (C) *Glast-Cre Arap1^tm1c/tm1c^* retinas also demonstrated frequent gaps between AJ complexes (indicateds by the white arrowheads) alternating with regions of normal AJ complexes (black arrowheads) and loss of linear arrangement. (D) Littermate *Glast-Cre Arap1^tm1c/+^* retinas did not demonstrate abnormalities in the external limiting membrane (ELM). (E) *Rlbp1-Cre Arap1^tm1c/tm1c^* retinas also demonstrated abnormal ELM junction structure with significant gaps between AJs, although linear arrangement was relatively preserved. (F) These abnormalities were not observed in *Rlbp1-Cre Arap1^tm1c/+^* littermate retinas. Images were taken at 4300× magnification.

Despite the lack of any discernible phenotype by retinal imaging and histology, *Glast-Cre Arap1^tm1c/tm1c^* retinas have structural abnormalities of Müller glia, as detected by transmission electron microscopy. Compared to that in WT retinas (Fig. 4B), the external limiting membrane (ELM) in *Arap1^−/−^*, *Glast-Cre Arap1^tm1c/tm1c^* and *Rlbp1-Cre Arap1^tm1c/tm1c^* retinas was discontinuous and disorganized (Fig. 4A,C,E). Gaps between adherens junction (AJ) complexes and loss of linear arrangement were common in both germline and conditional mutant retinas. Littermate *Glast-Cre Arap1^tm1c/+^* and *Rlbp1-Cre Arap1^tm1c/+^* retinas demonstrated structurally normal ELM (Fig. 4D,F).

Although not documented in *Rlbp1-Cre* or *Glast-Cre* mice, Cre toxicity in the noninduced *Vmd2-Cre* line has been debated (He et al., 2014; Iacovelli et al., 2011). To account for potential Cre cytotoxicity in all cKO lines, we analyzed *Rlbp1-Cre Arap1^tm1c/+^*, *Glast-Cre Arap1^tm1c/+^* and *Vmd2-Cre Arap1^tm1c/+^* littermate retinas with fundus imaging, histology and OCT measurement. None of the *Cre* heterozygotes demonstrated significant differences in fundus appearance, histological analysis and OCT analysis from WT mice (Fig. S5). Furthermore, analysis of *Vmd2-Cre Arap1^tm1c/tm1c^* mice was carried out at 1 month of age as no Cre-related degeneration has been documented by this time point (He et al., 2014).

### Human retinal ARAP1 interactome

**Fig. 5.**
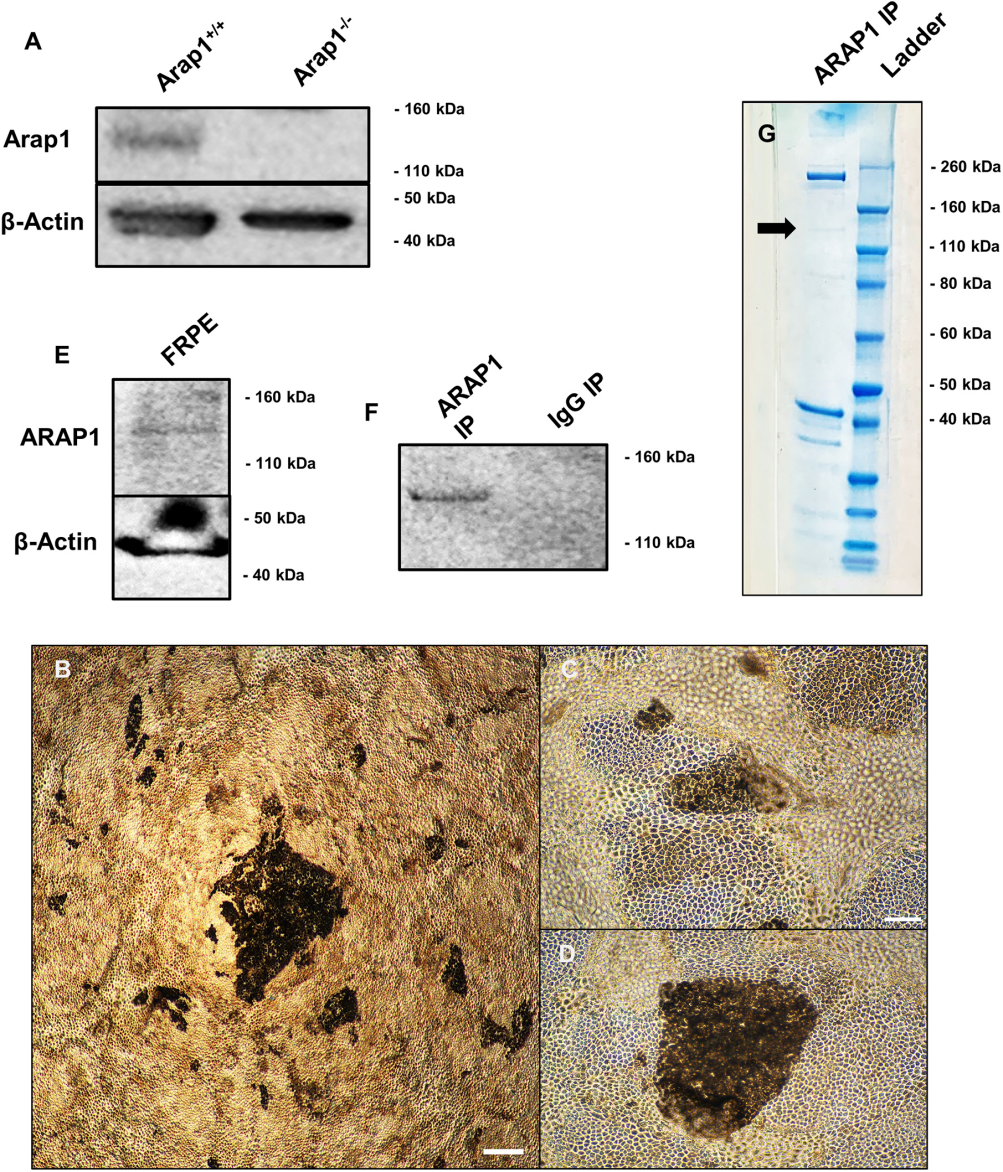
**ARAP1 co-immunoprecipitation.** (A) Western blot analysis validated that the anti-Arap1 antibody detected a band at ∼136 kD in *Arap1^+/+^*, but not *Arap1^−/−^* mouse liver lysate. β-actin was included as an endogenous control. (B-D) Fetal RPEs (FRPEs) were harvested from donor tissue and grown in culture. Cells were lysed with NP-40 lysis buffer when mature, as defined by pigmentation and hexagonal ‘cobblestone’ morphology (passage 1, culture day 98). (E,F) Immunoprecipitation of FRPE lysate with anti-ARAP1 was performed with a parallel goat IgG control immunoprecipitation. Immunoprecipitates were analyzed with western blot analysis along with FRPE lysate. A band was detected at ∼136 kDa in both FRPE lysate (E) and anti-ARAP1 immunoprecipitate, but not the control goat IgG immunoprecipitate (F). (G) Coomassie Blue analysis of the anti-ARAP1 immunoprecipitation, with a faint blue band at ∼136 kDa (arrow). Uncropped blots are shown in Fig. S6. Scale bars: 300 μm (B), 100 μm (C). Images were taken at 4× (B) and 10× magnification (C,D).

After determining that Arap1 is essential in RPE cells for photoreceptor survival, we sought to understand the specific function of this protein in these cells. To elucidate potential cellular processes in which Arap1 is involved, co-immunoprecipitation of ARAP1 and its binding partners was performed on cultured human fetal RPE (FRPE) cells. The antibody, previously validated for western blot detection and immunoprecipitation of ARAP1, was able to detect Arap1 in WT mouse liver lysate, but not *Arap1^−/−^* liver lysate (Segeletz et al., 2018). A band at ∼136 kDa was observed, consistent with Arap1 isoform 3 (Fig. 5A). Additional bands were observed in the WT lysates, likely due to non-specific binding (Fig. S6A). To ensure that this antibody bound human ARAP1, we obtained fetal donor eyes and meticulously dissected RPE tissue for culture. To ensure that protein expression patterns were similar to those in *in vivo* RPE, cultures were maintained until they adopted physical characteristics of maturity as defined previously by pigmentation and ‘cobblestone’ hexagonal morphology (Fig. 5B-D) (Adijanto and Philp, 2014; Al-Ani et al., 2020). Western blot analysis was performed on FRPE lysate, which detected a band at the same molecular mass (Fig. 5E).

To ensure that candidate binding partners of ARAP1, detected by mass spectrometry, were not precipitated due to non-specific binding to the magnetic Protein G beads, FRPE lysates were pre-cleared with beads, and bead–antibody complexes were cross-linked. To control for non-specific binding due to the antibody itself, non-specific goat IgG immunoprecipitations were carried out in parallel to goat anti-ARAP1 immunoprecipitations. On western blot analysis using FRPE cell lysate, ARAP1 was detected at 136 kD in the anti-ARAP1 immunoprecipitate, but not control anti-IgG immunoprecipitate (Fig. 5F). We analyzed the anti-ARAP1 immunoprecipitate post-gel electrophoresis with Coomassie Blue staining to ensure that there was sufficient protein for mass spectrometry analysis. Coomassie Blue analysis yielded a blue band at ∼136 kD (Fig. 5G).

Upon initial liquid chromatography–tandem mass spectrometry (LC-MS/MS) analysis, we identified 816 proteins with a threshold of 95%. Further stratification was performed with the following criteria: percentage sequence coverage >4%, number of unique peptides >3, relative abundance ratio (anti-ARAP1 immunoprecipitation/control IgG immunoprecipitation) >5, and significant protein detection in a minimum of three of five biological replicates. Post-stratification, ∼150 candidate binding partners remained. LC-MS/MS detected numerous proteins involved in actin cytoskeletal management that have been detected in the past, including members of the actin-related protein 2/3 (ARP2/3) complex family and F-actin capping protein complex (Segeletz et al., 2018). Novel interactants in the composition of the actin cytoskeleton were identified as well. These included components of microfilaments (ezrin, calponin-3, moesin) and intermediate filaments (desmocollin-1) essential for the formation of the RPE cytoskeleton as well as F-actin regulators (ankycorbin) (Tarau et al., 2019). Many members of the myosin family were identified (MYO6, MYO7A, MYH9, MYH10) (Tarau et al., 2019). These results align with previously established roles of ARAP1 in cytoskeletal coordination (Segeletz et al., 2018; Tanna et al., 2019).

Many established components of the RPE phagocytic machinery were identified, such as unconventional myosin-7a (MYO7A). Mutations in MYO7A have been linked to Usher syndrome, a condition denoted by hearing loss and RP, and defects in RPE phagocytosis of rod OS (Soni et al., 2005). Subunits of V-type proton ATPase (V-ATPase), a lysosome-associated protein, were identified. V-ATPase has been shown to be an essential component of RPE phagocytic machinery and subsequent photoreceptor maintenance (Nuckels et al., 2009; Shine et al., 2014). Components and known interactors of non-muscle myosin type II (NM2) were found, such as MYL6B, MYLK, MYH9, MYH10 and CTNNB1. NM2 is an essential participant of RPE phagocytic machinery through its interactions with Mer tyrosine kinase (MerTK) (Strick et al., 2009). Other identified proteins implicated in phagocytosis, but with no established role in RPE, include members of the coronin family (coronin-1, coronin-2) (Yan et al., 2005).

Beyond possible phagocytic interactions, participants of the endocytic pathway were identified, such as the adaptor protein-2 (AP-2) complex. ARAP1 has, in the past, been implicated to regulate the endocytosis of EGFR and interact with another clathrin-associated member of the AP family, AP-3 (Segeletz et al., 2018; Yoon et al., 2008). Consistent with this possibility, many elements of clathrin (CLTA, CLTB, CLTC) and clathrin adaptors were identified (CLINT1, EPN1, HIP1, STON2) (Metzler et al., 2001; Yao et al., 2006). Unconventional myosin-6 (MYO6) and many of its formerly validated interactants were precipitated. These interactants include cargo adaptors DAB2 and TOM1 and the entirety of the DOCK7-induced septin displacement (DISP) complex (MYO6, DOCK7, LRCH3). These results are summarized in Table 1.

**Table 1.**
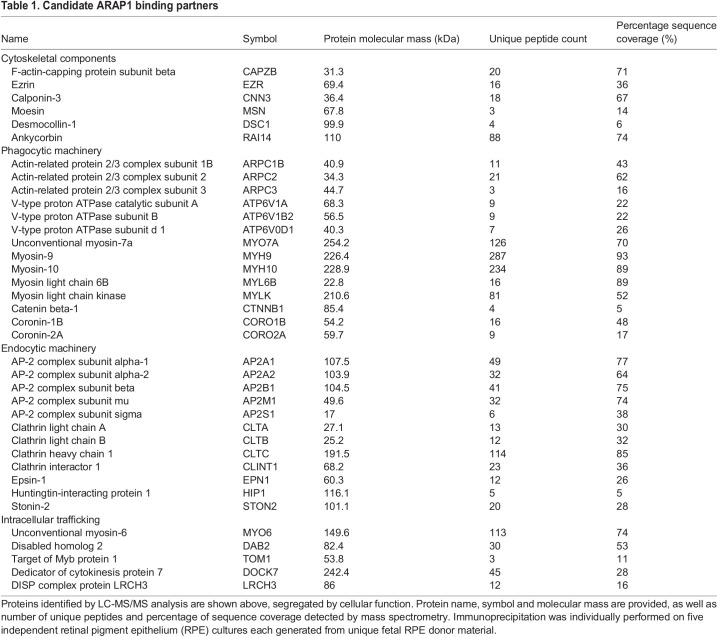
Candidate ARAP1 binding partners

Several established ARAP1 binding partners were immunoprecipitated but just missed criteria for inclusion after stratification. SH3 domain-containing kinase-binding protein 1 (CIN85; also known as SH3KBP1) was detected in two of five experimental samples and in none of the controls. CIN85 has been formerly validated as an ARAP1 interactor by the yeast two-hybrid system and LC-MS/MS analysis (Havrylov et al., 2009; Segeletz et al., 2018; Yoon et al., 2011). Cell division control protein 42 (Cdc42) was detected in three of five experimental samples and was significantly enriched compared to the control immunoprecipitation. However, only one unique peptide was identified, preventing its inclusion. Ras-related C3 botulinum toxin substrate 1 (RAC1) was also identified in three of five experimental samples but lacked the enrichment required for its inclusion. Both CDC42 and RAC1 were near absent in control samples. CDC42 and RAC1 are key drivers of actin polymerization in RPE phagocytosis (Kevany and Palczewski, 2010). Notable proteins failing inclusion criteria are summarized in Table S2.

### *Arap1^−/−^* mice demonstrate RPE phagocytic defect

**Fig. 6.**
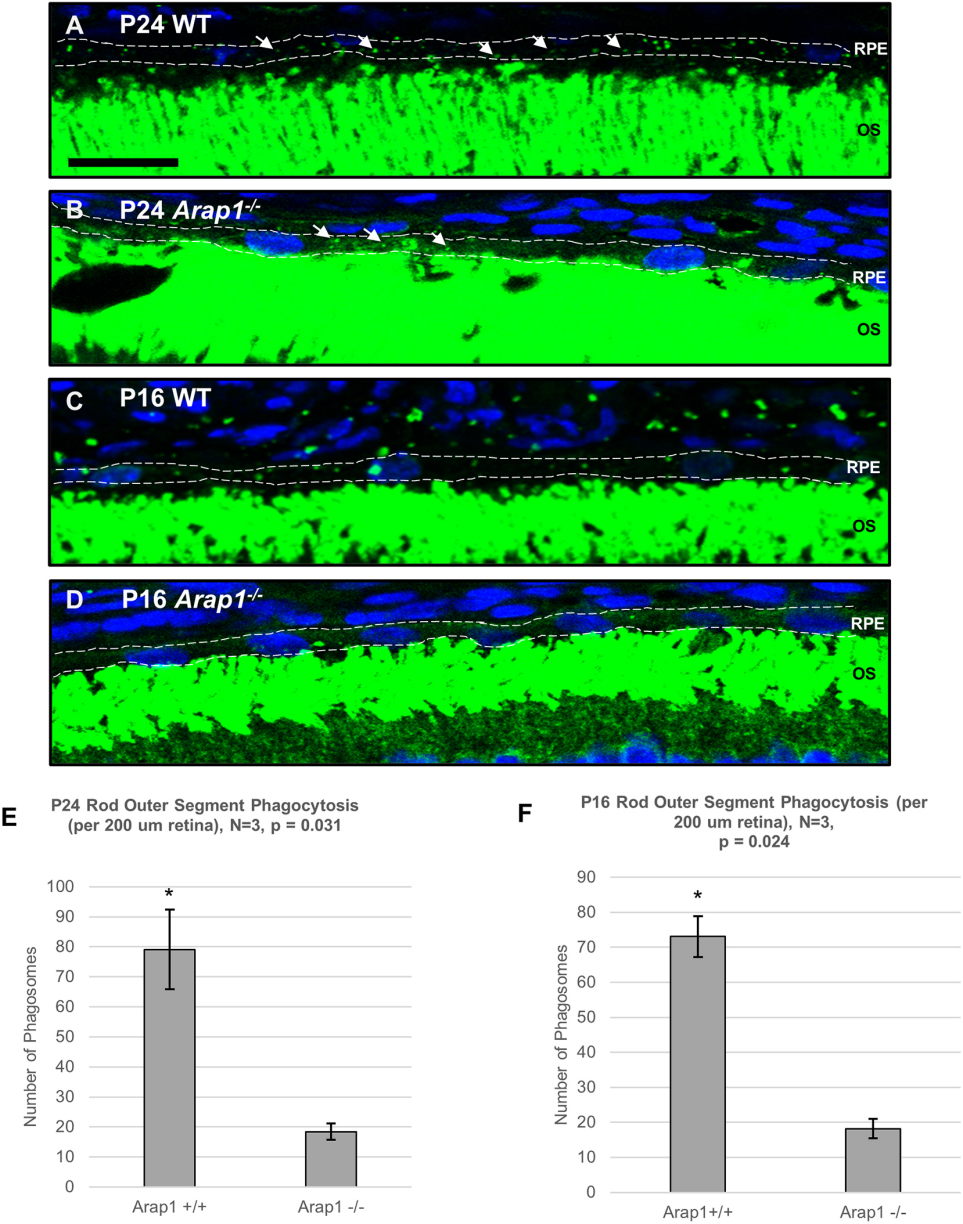
**Reduction of RPE rod phagocytosis in *Arap1*^−/−^ retinas.** (A-D) Immunohistochemistry using anti-rhodopsin (green) and anti-M- and anti-L-opsin (red, not pictured, shown in Fig. S6) was performed to quantify rod and cone RPE phagosomes, respectively, with DAPI counterstaining (blue) to visualize the RPE nuclei. Only merged images of anti-rhodopsin immunosignal and DAPI staining are shown. (A,B) Rod phagosomes (white arrows) were counted in eyes sectioned at P24 in both *Arap1^−/−^* mice and WT littermates. Only phagosomes present in the RPE layer were counted (first monolayer of nuclei). Examples of counted boundaries are shown (white dashed lines) and in Fig. S8. Scale bar: 20 μm. (E) *Arap1^−/−^* retinas demonstrated reduced numbers of rod phagosomes (18.4±2.7 per 200 μm retina) compared to WT littermates (79.1±13.3 per 200 μm retina). (C,D) Phagosomes were also quantified in P16 *Arap1^−/−^* and WT littermates. (F) *Arap1^−/−^* retinas at P16 also demonstrated reduced numbers of rod phagosomes (18.2±2.8 per 200 μm retina) compared to their WT littermates (73.1±5.8 per 200 μm retina). Uncropped low-power images of the same sections with RPE boundaries defined are shown in Fig. S8. Quantifications of cone phagosomes are shown in Fig. S7. *n*=3 for each group, tissue was collected from three different animals of each respective genotype, average values represent the mean, error bars represent s.e.m. Significance calculated by two-tailed, unpaired Student's *t*-test, *P<0.05.

As LC-MS/MS analysis revealed a significant number of interactants related to phagocytic machinery, we evaluated the role of *Arap1* in OS phagocytosis. Retinal sections of *Arap1^−/−^* and *Arap1^+/+^* littermates were stained with fluorescent antibodies against rhodopsin (green) for rod quantification and M- and L-opsins (red) for cone quantification, with 4′,6-diamidino-2-phenylindole (DAPI) counterstaining (blue) to better visualize the RPE layer. To account for circadian variation in RPE phagocytosis, mouse sacrifice was standardized to 1.5 h after light onset (Bobu and Hicks, 2009). At P24, *Arap1^−/−^* mice demonstrated a reduction in rod phagosomes (18.4±2.7 per 200 μm retina) compared to their WT littermates (79.1±13.3 per 200 μm retina) (Fig. 6A,B,E). Cone phagosomes, however, were comparable between the two groups (*Arap1^−/−^*: 13.2±3.7 per 2 mm retina, 29.7±9.2 per retinal section; *Arap1*^+/+^: 10.5±1.7 per 2 mm retina, 38.3±9.2 per retinal section) (Fig. S7E-H; examples shown in Fig. S7A -D′).

However, *Arap1^−/−^* retinas already experienced significant photoreceptor degradation by P24. As such, OS phagocytosis of pups at eye opening (P16) was also quantified to confirm that loss of phagosomes was due to an intrinsic phagocytic defect. Analysis of P16 retinas revealed markedly less degeneration. Measurement of P16 RPE phagocytosis revealed a similar reduction in rod phagosomes in knockout retinas compared to *Arap1^+/+^* littermate retinas (*Arap1^−/−^*: 18.2±2.8 per 200 μm retina; *Arap1^+/+^*: 73.1±5.8 per 200 μm retina) (Fig. 6C,D,F). As was seen in the P24 quantification, no significant difference was noted in cone phagosomes (*Arap1^−/−^*:5.7±2.0 per 2 mm retina, 15.0±7.1 per retinal section; *Arap1*^+/+^: 7.3±2.6 per 2 mm retina, 20.0±5.5 per retinal section) (Fig. S7F,H). Rarely, phagosome-like signal was observed in the choroid (Fig. 6C). However, this signal was not considered for phagosome counts, and only phagosome signal present in the RPE monolayer was quantified (Fig. 6A-D, white dashed lines). Low-power images of Fig. 6A-D are shown in Fig. S8.

## DISCUSSION

The findings of our study reveal that *Arap1* expression in RPE is essential for photoreceptor survival due to its function in RPE phagocytosis, possibly as a critical interactant of the RPE phagocytic pathway. *Rlbp1-Cre Arap^tm1c/tm1c^* and *Vmd2-Cre Arap1^tm1c/tm1c^* mice recapitulate the phenotype originally observed in *Arap1^−/−^* mice as confirmed by histopathology, OCT and fundus analysis. The milder degeneration observed in the *Rlbp1-Cre Arap^tm1c/tm1c^* mice is explained by the relative reduction in RPE Cre function in this line, possibly secondary to reduced *Cre* expression by the *Rlbp1* promoter in RPE cells (Fig. 2E). The mechanism underlying these degenerative changes is the significant reduction in RPE rod OS phagocytosis secondary to *Arap1* loss, as confirmed with immunohistochemical studies. Rod phagosomes were generated by *Arap1^−/−^* RPE at ∼25% of the rate of *Arap1^+/+^* RPE (Fig. 6E,F). It is somewhat unexpected that rod phagocytosis was impeded, whereas cone phagocytosis was comparable to that in controls. However, these abnormalities in phagocytosis do mirror the functional impediments we saw on ERG analysis of *Arap1^−/−^* mice previously, such that scotopic impairment appeared sooner and with greater severity than photopic impairment (Moshiri et al., 2017). Furthermore, previous studies suggest that phagocytosis of rod and cone OSs by RPE may be differentially regulated (Ying et al., 2018). However, further analysis will be required to definitively establish that *Arap1* loss results in a rod-specific phagocytosis defect. Despite the phagocytic defect, we did not observe any generalized RPE loss or degeneration. In our original publication, we observed hyperpigmentation at the level of the RPE fundoscopically, likely secondary to retinal thinning (Moshiri et al., 2017).

Although *Arap1* is expressed in Müller glia, its function does not seem to be essential for maintaining photoreceptors, as shown by analysis of *Glast-Cre Arap1^tm1c/tm1c^* mice. This could be explained by the possibility that Arap1 is expressed at a low level in Müller glia, consistent with the LacZ staining pattern we observed (Fig. 1E) as well as the β-galactosidase immunohistochemistry we published previously. Müller glia do, however, require *Arap1* for structural function. Ultrastructural analysis of *Glast-Cre Arap1^tm1c/tm1c^*, *Rlbp1-Cre Arap1^tm1c/tm1c^* and *Arap1^−/−^* retinas revealed discontinuity of the ELM absent in WT and -*Cre Arap1^tm1c/+^* retinas. The ELM is a network-like series of junctional complexes between Müller glia and photoreceptors essential for maintaining structural integrity of the retina (Omri et al., 2010). Adherens junctions (AJs) are composed of E-cadherin, p120-catenin, β-catenin and α-catenin (Hartsock and Nelson, 2008). As reviewed by Hartsock and Nelson (2008), p120-catenin has been shown to interact with Rho family GTPases to regulate actin cytoskeletal dynamics. Given its ability to regulate Rho GTPases, ARAP1 may regulate AJ–cytoskeleton communication in some capacity.

LC-MS/MS analysis of anti-ARAP1 immunoprecipitate revealed several avenues of interest in uncovering both the specific function of ARAP1 in RPE cells and mechanisms of RPE phagocytosis. Considering the diversity of the functional domains of ARAP1, it is not surprising that several different processes are implicated by the results of mass spectrometry analysis. Given that interruption of photopigment (11-cis retinaldehyde) recycling can cause RP, it is noteworthy that no proteins related to this pathway were identified (Ferrari et al., 2011).

Many of the proteins identified were involved in the clathrin-mediated endocytic pathway, such as the AP-2 complex, clathrin elements, endocytic adaptors, and MYO6 and its interactants (Boucrot et al., 2010; O'Loughlin et al., 2018). The inclusion of DAB2 is particularly interesting as it has been shown to bind both the AP-2 complex and MYO6, bridging endocytosis and actin cytoskeletal regulation – two processes in which ARAP1 has been implicated (Morris et al., 2002). DAB2 has also been shown to bind CIN85, an established ARAP1 interactant, to target CIN85 to clathrin-coat assembly for EGFR endocytosis (Kowanetz et al., 2003). CIN85, AP-1, AP-2 and AP-3 complexes, clathrin and ARAP1 have been shown to colocalize during EGFR endocytosis. (Yoon et al., 2011) Furthermore, CIN85/ARAP1 likely affect targeting of the pre-early endosome, the endocytic stage at which DAB2 and MYO6 are active (Yoon et al., 2011; Hasson, 2003). Although CIN85 was unable to meet inclusion criteria, it is a notable protein identified in our LC-MS/MS analysis (Table S2).

Our LC-MS/MS analysis revealed numerous interactants of RPE phagocytosis, which is mechanistically similar to the Fcγ receptor (FcγR) phagocytosis system as reviewed by Kevany and Palczewski (2010). Accordingly, RPE phagocytosis consists of several sequential steps: recognition, binding, internalization, intracellular trafficking and digestion. Components of RPE phagocytic machinery have been identified previously. MerTK and its ligands Gas6 and Protein S are involved in the internalization of shed OS membranes, adhesion receptor αvβ5 integrin regulates recognition/binding, and scavenger receptor CD36 facilitates internalization (Kevany and Palczewski, 2010). Receptor regulation of RPE phagocytosis is complex, as elucidated elements of their respective and often independent signaling pathways has revealed. PI3K and Akt have been shown to regulate internalization and the recruitment of F-actin and NM2 (Bulloj et al., 2013). Rac1 has been shown to be essential for F-actin recruitment downstream of αvβ5 integrin signaling, independent of MerTK (Mao and Finnemann, 2012). RhoA-associated kinase activity is sufficient to restore RPE phagocytosis in MerTK-deficient RPE (Mao and Finnemann, 2021).

Beyond receptor interactions, several cytoplasmic proteins have also been linked to RPE phagocytosis. Knockout of myosin-7 and annexin A2 leaves engulfed phagosomes localized in the apical region of RPE cells, while WT RPE traffics phagosomes to the basal region (Kevany and Palczewski, 2010; Law et al., 2009). Even further downstream in the phagocytic pathway, knockout of melanoregulin, a putative membrane fusion regulator protein, demonstrates a normal number of phagosomes generated but without normal decline, suggesting impairment in phagosome digestion post-engulfment (Kevany and Palczewski, 2010).

In our list of candidate Arap1 binding partners, many proteins identified have clear roles in the cytoskeletal dynamics of RPE phagocytosis. The ARP2/3 complex and β-catenin play essential roles in actin polymerization and organization during phagocytosis (Kevany and Palczewski, 2010). Moreover, numerous components and interactants of NM2 were identified: MYH9, MYH10, MYL6B and MYLK (Betapudi, 2014; Fuertes-Alvarez et al., 2018; Hirota et al., 2010; Xie et al., 2018). NM2 has been shown to be recruited downstream of MerTK signaling to the nascent phagosome and is essential for RPE engulfment of OS. Following that observation, it was hypothesized that NM2 and MerTK may be members of a protein complex that governs RPE phagocytic engulfment (Strick et al., 2009). Motor proteins of both isoforms NM2A and NM2B (MYH9 and MYH10, respectively) were identified. Although depletion of either isoform can impede RPE phagocytosis, NM2A has been identified as a specific interactant of MerTK through immunoprecipitation and mass spectrometry analysis (Strick et al., 2009). This result is in line with the understanding that NM2 mediates cellular protrusion, a process essential for the formation of phagocytic pseudopodia (Castellano et al., 2001). Interestingly, NM2 also plays an essential role in stabilizing AJ integrity (Heuzé et al., 2019; Smutny et al., 2010). Recalling the ELM abnormalities seen in *Arap1^−/−^*- and *Glast*- and *Rlbp1-Cre Arap^tm1c/tm1c^* mice, involvement of NM2 could possibly explain the phagocytic defect we see in RPE cells, and also the abnormal AJs seen in Müller glia. Similar to their dynamic in RPE phagocytosis, NM2A and NM2B play complementary roles in AJ formation and maintenance (Heuzé et al., 2019). Although interaction between ARAP1 and NM2 has yet to be documented, another Arf-directed GAP family member, ASAP1, has previously been shown to bind NM2A (Tanna et al., 2019).

Another notable set of identified proteins are LRCH3, MYO6 and DOCK7, which together compose the DISP complex. The DISP complex has been shown to regulate the organization of septins, a family of filament-forming, GTP-binding proteins essential for phagosome formation in FcγR phagocytosis (Huang et al., 2008; O'Loughlin et al., 2018). Furthermore, members of the septin family are essential in scaffolding for the formation of NM2 fibers (Joo et al., 2007).

Consistent with prior publications (Yoon et al., 2011; Miura et al., 2002), our preliminary explorations into the RPE interactome of ARAP1 includes participants in endocytosis and cytoskeletal dynamics. This work suggests a role for ARAP1 in RPE phagocytosis, as evidenced by the OS phagocytosis defect observed in *Arap1^−/−^* mice. Further analysis will be needed to discern whether this defect is isolated to rod OS membrane clearance. Although numerous elements of RPE phagocytic machinery were identified in our LC-MS/MS analysis, further investigation is necessary to confirm the validity of these candidate interactors and elucidate the specific mechanism of ARAP1 in RPE phagocytosis.

## MATERIALS AND METHODS

### Animals

This study was conducted according to a protocol that was approved by the Institutional Animal Care and Use Committee at UC Davis. Ages of animals used for each assay are detailed in the legend of the relevant figure. Both male and female mice were used in analyses.

### Generation of *Arap1 Tyr^c-2J^*/*Tyr^c-2J^* mice

*Arap1* germline knockout mice (*Mus musculus*) were generated by the UC Davis Mouse Biology Program as previously described (Moshiri et al., 2017). B6(Cg)-Tyrc-2J/J mice were acquired from The Jackson Laboratory (stock #000058) and bred to *Arap1*^−/−^ germline knockouts. The progeny were then bred with B6(Cg)-Tyrc-2J/J to obtain *Arap1*^+/−^ *Tyr^c-2J^*/*Tyr^c-2J^*, which were used as founders for this strain, colloquially referred to as albino *Arap1* germline knockouts.

### Generation of *Arap1* conditional mouse strains

Floxed *Arap1* conditional mice (*Mus musculus*) on the C57BL/6N background were generated by the UC Davis Mouse Biology Program using the knockout-first, promoter-driven selection cassette strategy to generate tm1c mice with cKO potential (Pettitt et al., 2009). These founders were bred with C57BL/6J mice, and colony founders were confirmed to be free of the *Crb1* mutation (*rd8*) by PCR – all mice in this study were confirmed to be free of *rd8*. Three independent strains were then established for the cKO of *Arap1* using established *Cre* lines: Tg(Slc1a3-cre/ERT)1Nat (referred to as *Glast-Cre*, from The Jackson Laboratory, stock #012586); Tg(BEST1-cre)1Jdun (referred to as *Vmd2-Cre*, from The Jackson Laboratory, stock #017557) and Tg(Rlbp1-Cre/ERT) (referred to as *Rlbp1-Cre*, generously provided by Dr Edward Levine) (Pollak et al., 2013; Vázquez-Chona et al., 2009). For each colony, male *Arap1^tm1c/+^ Cre*+ mice were bred with female *Arap1^tm1c/tm1c^* mice to obtain progeny for our study (*Arap1^tm1c/+^ Cre*+ and *Arap1^tm1c/tm1c^ Cre*+). For both *Glast-Cre* and *Rlbp1-Cre*, Cre activity was induced with intraperitoneal injection of tamoxifen (75 mg/kg of body weight in corn oil) once per day for five consecutive days beginning from P30. Induction at P30 was selected as we previously observed retinal histogenesis is completed at this age, and *Arap1* germline knockouts began to degenerate at 3-4 weeks postnatal. This ensured that any degenerative changes observed in these mice suggested a defect in retinal maintenance rather than retinal development. Given the silencing of *Cre* expression known to affect the *Vmd2-Cre* line, *Vmd2-Cre* breeders, which produced progeny with minimal or no *Cre* expression, were removed from the breeding program, as were their progeny (Iacovelli et al., 2011; He et al., 2014). Breeders were regularly bred onto an Ai9 to assess Cre function. Expression was limited to the RPE in *Vmd2-Cre*, Müller glia in *Glast-Cre* and RPE/Müller glia in *Rlbp1-Cre* lines. Global Cre function was never observed in any animals crossed onto the Ai9 background.

### Histology

For cryoembedding, eyes were enucleated, promptly fixed with 4% PFA for 1 h at ambient temperature and then stepwise dehydrated in 10% sucrose, 20% sucrose and 30% sucrose in PBS. The eyes were embedded in Tissue Plus OCT compound (Fisher Scientific) then snap frozen in a dry ice–ethanol bath. Sections (12 μm) were obtained using a cryostat (LEICA CM3050; Leica, Wetzlar, Hesse, Germany).

For paraffin embedding, eyes were processed as described in Sun et al. (2015). Briefly, eyes were enucleated and snap frozen in dry ice-cooled propane. The eyes were then stepwise fixed with 3% acetic acid in 97% methanol by storing at −80°C for 7 days, −20°C overnight and finally in ambient temperature for 2 days. The eyes were then processed into paraffin, beginning with dehydration in 100% ethanol (two changes of 100% ethanol; 1 h each), replacement in xylene (two changes; 15 min each) and then 60°C paraffin (three changes; 1 h each). Sections (5 μm) were obtained using a Leica RM2125Rt microtome.

### Immunohistochemistry

Cryosections were washed in 1× PBS (three times; 5 min each) and blocked for 1 h at ambient temperature with blocking solution [4% bovine serum albumin (BSA) in 10 mM Tris-HCl pH 7.4, 10 mM MgCl_2_ and 0.5% v/v Tween 20]. Primary antibodies were then diluted in blocking buffer as specified and applied to the sections overnight at 4°C. Before corresponding Alexa Fluor-conjugated secondary antibodies were added for 1 h at ambient temperature, the cryosections were washed in PBS (three times; 5 min each). Then, 1 μg/ml DAPI was added to the cryosections at ambient temperature for 5 min, and the slides were washed in PBS (three times; 5 min each). Cryosections were then coverslipped with FluorSave Reagent (Millipore).

For paraffin sections, sections were deparaffinized using xylene (three times; 5 min each). The tissue sections were then stepwise hydrated by submersion in 100% ethanol (two times; 5 min each), 95% ethanol (5 min), 75% ethanol (5 min) and distilled water. Heat-induced epitope retrieval was then performed with 1 mM EDTA, pH 8.0, and the tissue sections were blocked for 30 min in blocking solution in ambient temperature. Similarly, primary antibodies, DAPI and coverslipping was done as described in the cryosection methods.

### TUNEL

Detection of apoptotic cells was determined using an ApopTag^®^ Fluorescein In Situ Apoptosis Detection Kit (Sigma-Aldrich). Paraffin sections were deparaffinized and hydrated as described, and then treated with proteinase K (20 μg/ml) at ambient temperature. The tissue sections were then washed with PBS (two times; 2 min each). The tissue sections were equilibrated, and TdT enzyme was applied for 1 h at 37°C. The reaction was stopped with stop buffer, and the sections were washed in PBS (three times; 1 min each). The anti-digoxigenin conjugate was applied for 30 min at ambient temperature, followed by DAPI and coverslipping as described. Finally, the slides were coverslipped with FluorSave Reagent (EMD Millipore). TdT enzyme, equilibration buffer, stop buffer and anti-digoxigenin conjugate were provided with the kit. Quantification was performed per high-power field at 40× magnification.

### H&E staining

Sections were deparaffinized using xylene (three times; 5 min each), and then stepwise hydrated by submersion in 100% ethanol (two times; 5 min each), 95% ethanol (5 min), 75% ethanol (5 min) and distilled water (5 min). The sections were stained with Hematoxylin for 10 min, stained with Eosin for 5 min and dehydrated in reverse of the hydration procedure described beginning from 75% ethanol. They were then coverslipped with VectaShield (Vector Laboratories).

### β-galactosidase histochemistry

Cryosections were washed in 1× PBS (three times; 5 min each) and incubated in X-gal-containing solution (1 mg/ml X-gal, 5 mM potassium ferricyanide, 5 mM potassium ferrocyanide and 2 mM MgCl_2_ in 1× PBS) for 24 h in a 37°C incubator. The cryosections were then washed in 1× PBS (three times; 5 min each) and coverslipped with VectaShield.

### Ocular imaging: fundus photography and OCT

Mice were sedated with intraperitoneal injections with a combination of ketamine (50 mg/kg) and dexmedetomidine (0.25-0.5 mg/kg). The eyes were dilated with 2.5% phenylephrine hydrochloride (Akorn, Lake Forest, IL, USA) and tropicamide (Bausch & Lomb, Tampa, FL, USA), and then lubricated with GenTeal (Alcon Laboratories, Fort Worth, TX, USA). Fundus images were obtained with StreamPix 5 using a Micron III (Phoenix Research Laboratories, Pleasanton, CA, USA).

OCT was performed using an Envisu R2200 SDOIS Imaging System (Bioptigen, Morrisville, NC, USA) and analyzed using InVivoVue 2.4.35.

### Scanning electron microscopy

Eyes were fixed in 2.5% glutaraldehyde and 2% paraformaldehyde in 0.1 M sodium cacodylate buffer (all from Electron Microscopy Sciences) overnight with gentle rocking at 4°C, washed with 0.1 M cacodylate buffer and post-fixed in 1% osmium tetroxide for 2 h at ambient temperature. The eyes were then dehydrated in a graded ethanol series, further dehydrated in propylene oxide and embedded in Epon epoxy resin. Semi-thin (1 μm) and ultra-thin (100 nm) sections were cut with a Leica EM UC6 ultramicrotome, and the latter were collected on pioloform-coated (Ted Pella) one-hole slot grids. Sections were contrasted with Reynolds lead citrate and 8% uranyl acetate in 50% ethanol and imaged on a Philips CM120 electron microscope equipped with an AMT BioSprint side-mounted digital camera and AMT Capture Engine software.

### ERG

ERG analysis was performed on both eyes of mice prior to euthanasia (LKC Bigshot; LKC Technologies, Gaithersburg, MD, USA). Mice were dark adapted overnight prior to ERG analysis. Mice were anesthetized intraperitoneally with a cocktail of ketamine (100 mg/ml to 1 ml) and xylazine (100 mg/ml to 0.1 ml) at a dosage of 0.1 ml/10 g diluted 1:10 in sterile saline. Post-anesthesia, mice were placed upon a heating pad held at 38°C, and one eye was pharmacologically dilated with 1% tropicamide and 2.5% phenylephrine eye drops. Topical anesthesia with proparacaine eye drops was also applied. After lubrication with 1% methylcellulose, mouse contact lens electrodes (LKC Technologies) were placed on each eye, needle reference electrodes were placed in each cheek, and a ground needle electrode was placed at the base of the tail. Full-field ERG was used to test scotopic retinal function (white flashes of increasing stimulus intensity, cd*s/m^2^) and, after 10 min of light adaptation (30 cd/m^2^), photopic retinal function using a similar series of white stimuli generated by a Ganzfeld stimulator (UTAS Visual Diagnostic System with BigShot LED; LKC Technologies).

### Cell culture

Discarded de-identified human fetal eyes were collected for use in cell culture with permission from the UC Davis Institutional Review Board. Eyes were carefully dissected by removing the anterior segment, carefully removing the retina and peeling the RPE as a single sheet (monolayer) with the use of a dissecting microscope. Fetal RPEs were cultivated at 37°C under a humidified 5% CO_2_ atmosphere in 3D-Retinal Differentiation Media (3:1 Dulbecco's modified Eagle medium (DMEM)-F12, DMEM (high glucose) supplemented with 5% (v/v) fetal bovine serum (Atlanta Biologicals), 2 mM GlutaMAX (Thermo Fisher Scientific), 200 μM taurine, 1× penicillin/streptomycin (Thermo Fisher Scientific), 1:1000 chemically defined lipid supplement (Thermo Fisher Scientific) and 2% B27 supplement (Thermo Fisher Scientific), as described previously (Capowski et al., 2019). All cultures were free of contamination at the time of analysis. No cell lines were used. Only primary tissues were expanded for the time indicated. Eyes were harvested from de-identified human fetal tissues without any known chromosomal abnormalities or birth defects.

### Protein extraction

Differentiated cells were washed with ice-cold 1× PBS and incubated on ice for 10 min in ice-cold lysis buffer (25 mM Tris-HCl pH 7.4, 150 mM NaCl, 1 mM EDTA, 1% NP-40, 5% glycerol and protease inhibitors). Cells were then mechanically detached with a cell scraper and homogenized via a dounce homogenizer. For mouse liver samples, 250 μg of liver was homogenized in 750 μl of lysis buffer via a dounce homogenizer. Homogenate was centrifuged at 4°C at 14,000 ***g*** for 20 min, and supernatant was collected.

### Immunoprecipitation

Lysates were precleared on Protein G-magnetic beads (Thermo Fisher Scientific) for 1 h at 4°C. Protein G beads were incubated with antibodies for 1 h and subsequently cross-linked with 5 mM BS3 for 30 min at ambient temperature. Immunoprecipitation was performed with a Dynabeads™ Protein G Immunoprecipitation Kit (Thermo Fisher Scientific) with goat anti-ARAP1 (1:50, Abcam, ab5912) and normal goat IgG (1:50, R&D Systems, AB-108-C).

### Western blotting

Lysates and immunoprecipitates were loaded onto NuPAGE™ 4-12%, Bis-Tris, 1.5 mm gels (Thermo Fisher Scientific) and resolved. Gels were wet blotted onto polyvinylidene fluoride (PVDF) membranes and subsequently blocked with 5% BSA in TBST for 1 h at ambient temperature. Blots were incubated with goat anti-Arap1 IgG (1:2000, Abcam, ab5912) and rabbit anti-β-actin IgG (1:4000, Cell Signaling Technology, 4970S) overnight at 4°C. After incubation with horseradish peroxidase (HRP)-conjugated donkey anti-goat IgG (1:4000, Abcam, ab97110) and HRP-conjugated goat anti-rabbit IgG (1:4000, Cell Signaling Technology, 7074P2) for 1 h at ambient temperature, bands were detected with enhanced chemiluminescence western blotting detection reagents (Amersham). All antibodies used in this study are commercially available and were formerly validated by their respective companies. Catalogue numbers, dilutions and PMIDs of previous publications using all primary antibodies are summarized in Table S1.

### Sample preparation for liquid chromatography–mass spectrometry (LC-MS) analysis

Five independent fetal RPE samples (*n*=5) were cultured and included in this study. Proteins from each immunoprecipitate were subjected to tryptic digestion via suspension-trap (S-Trap) devices (ProtiFi). S-Trap is a powerful filter-aided sample preparation (FASP) method that consists of trapping acid aggregated proteins in a quartz filter prior to enzymatic proteolysis, and allows for reduction/alkylation/tryptic proteolysis all in one vessel. Specifically, proteins were resuspended in 50 µl solubilization buffer, consisting of 5% SDS, 50 mM triethyl ammonium bicarbonate, complete protease inhibitor cocktail (Roche), pH 7.5. Disulfide bonds were reduced with dithiothreitol and alkylated with iodoacetamide in 50 mM TEAB buffer. The enzymatic digestion occurred via a first addition of trypsin 1:100 enzyme: protein (wt/wt) for 4 h at 37°C, followed by a boost addition of trypsin using same wt/wt ratios for overnight digestion at 37°C. Peptides were eluted from S-Trap by sequential elution buffers of 100 mM TEAB, 0.5% formic acid, and 50% acetonitrile with 0.1% formic acid. The eluted tryptic peptides were dried in a vacuum centrifuge and re-constituted in 0.1% trifluoroacetic acid. These were subjected to LC-MS analysis.

### LC-MS

Peptides were resolved on a Thermo Fisher Scientific Dionex UltiMate 3000 RSLC system using a PepMap 75 μm×25 cm C18 column with 2 μm particle size (100 Å pores), heated to 40°C. A 0.6 μg of total peptide amount was injected for each sample, and separation was performed in a total run time of 90 min with a flow rate of 200 μl/min with mobile phases A (water/0.1% formic acid) and B (80%ACN/0.1% formic acid). Gradient elution was performed from 10% to 8% B over 3 min, from 8% to 46% B over 66 min, and from 46 to 99% B over 3 min, and, after holding at 99% B for 2 min, down to 2% B in 0.5 min, followed by equilibration for 15 min. Peptides were directly eluted into an Orbitrap Exploris 480 instrument (Thermo Fisher Scientific, Bremen, Germany). Spray voltage was set at 1.8 kV, funnel radio frequency level at 45 and heated capillary temperature at 275°C. The full MS resolution was set to 60,000 at m/z 200 and full MS automatic gain control (AGC) target was 300% with an injection time set to Auto. Mass range was set to 350-1500. AGC target value for fragment spectra was set at 200% with a resolution of 15,000, and injection time was set to Standard and Top40. Intensity threshold was kept at 5E3. Isolation width was set at 1.6 m/z, and normalized collision energy was set at 30%.

### Raw data processing

The LC-MS .raw files were processed with Proteome Discoverer 2.4 (Thermo Fisher Scientific) using the integrated SEQUEST engine. All data were searched against a target/decoy version of the human UniProt Reference Proteome without isoforms (21,074 entries). Peptide tolerance was set to 10 ppm, and fragment mass tolerance was set to 0.6 Da. Trypsin was specified as enzyme, cleaving after all lysine and arginine residues and allowing up to two missed cleavages. Carbamidomethylation of cysteine was specified as fixed modification, and protein N-terminal acetylation, oxidation of methionine, deamidation of asparagine and glutamine, and pyro-glutamate formation from glutamine were considered variable modifications, with a total of two variable modifications per peptide. False-discovery rates were limited to 1-5% for peptides.

### *In situ* phagosome quantification

Retinal cryosections were washed as described above and subsequently blocked at ambient temperature for 1 h. Sections were then incubated with mouse anti-rhodopsin IgG (1:1000, EMD Millipore, MABN15) and rabbit anti-L/M-opsin IgG (1:1000, EMD Millipore, AB5405) overnight at 4°C. Sections were washed with 1× PBS (three times; 5 min each) and incubated with donkey anti-mouse Alexa Fluor Plus 488 IgG (1:500, Thermo Fisher Scientific) and donkey anti-rabbit Alexa Fluor Plus 647 IgG (1:500, Thermo Fisher Scientific) at 37°C for 1 h. Slides were then stained for DAPI and washed as described above. Sections were coverslipped with FluorSave Reagent and imaged with an Olympus FV3000 Confocal Laser Scanning Microscope (Olympus Corporation, Shinjuku City, Tokyo, Japan). Rhodopsin- and cone opsin-positive phagosomes were quantified as previously described (Nandrot et al., 2007; Ying et al., 2018). Only phagosomes present in the RPE monolayer were quantified. This layer was judged to be the first layer of nuclei detected through DAPI staining beyond the photoreceptor OS. Example boundaries of counted sections are shown in Fig. S8.

### Statistical analysis

*N* was defined as biological replicates. Each experiment contained a minimum *N* of 3 as specified in the figure legends. Similar numbers of males and females were used in each group. Animals were segregated based on genotype and age. Technical replicates for each experiment were performed at a minimum of three times. However, owing to limitations in primary human fetal tissue, the co-immunoprecipitation and subsequent mass spectrometry analysis were only performed with *n*=5 samples in each group and one technical replicate. Comparisons between groups were performed using Student's *t*-test (two-tailed, unpaired). *P*<0.05 was considered statistically significant.

## Supplementary Material

Supplementary information
