# Supplementary material for: Arap1 loss causes retinal pigment epithelium phagocytic dysfunction and subsequent photoreceptor death
Source: Dis Model Mech. 2022 Jul 25;15(7):dmm049343. doi: 10.1242/dmm.049343 (PMC9346516; doi:10.1242/dmm.049343)
Supplement: Supplementary information [file dmm-15-049343-s1.pdf]

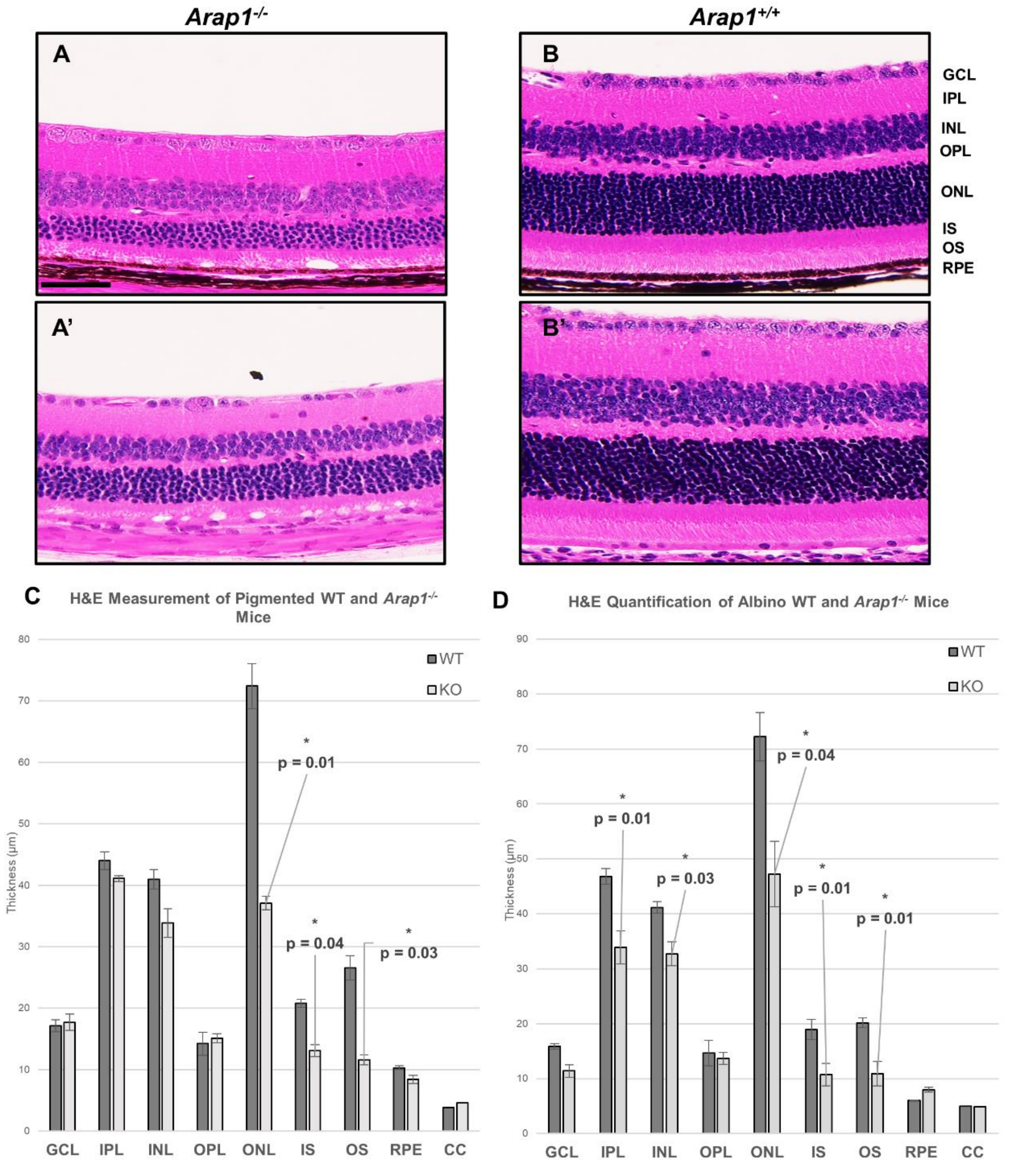

Fig. S1. Quantification of Retinal Layers in Pigmented and Albino Arap1$^{+/+}$ and Arap1$^{-/-}$ mice. Hematoxylin and eosin-stained sections of wild type and *Arap1*$^{-/-}$ mice collected from animals age postnatal day 42 of both albino (**A,A'**) and pigmented (**B,B'**) background were measured and quantified. In mutant animals of both the pigmented and albino groups, there was a statistically-significant reduction in the ONL, IS, and OS layers compared to their wild type counterparts (**C,D**). Additionally, there was significant degeneration in the IPL and INL layers in albino knockouts compared to albino wild type littermates (**D**). The ganglion cell layer (GCL), inner plexiform layer (IPL), inner nuclear layer (INL), outer plexiform layer (OPL), outer nuclear layer (ONL), inner segments (IS), outer segments (OS), and retinal

Disease Models & Mechanisms • Supplementary information

pigment epithelium (RPE) are labeled (**B**). Scale bar (**A**) represents 100 µm. N = 3 for each group, tissue was collected from 3 different animals of each respective genotype, significance calculated by two-tailed Student's t-test, p values shown in graph, average values represent the mean, error bars represent s.e.m.

Disease Models & Mechanisms • Supplementary information

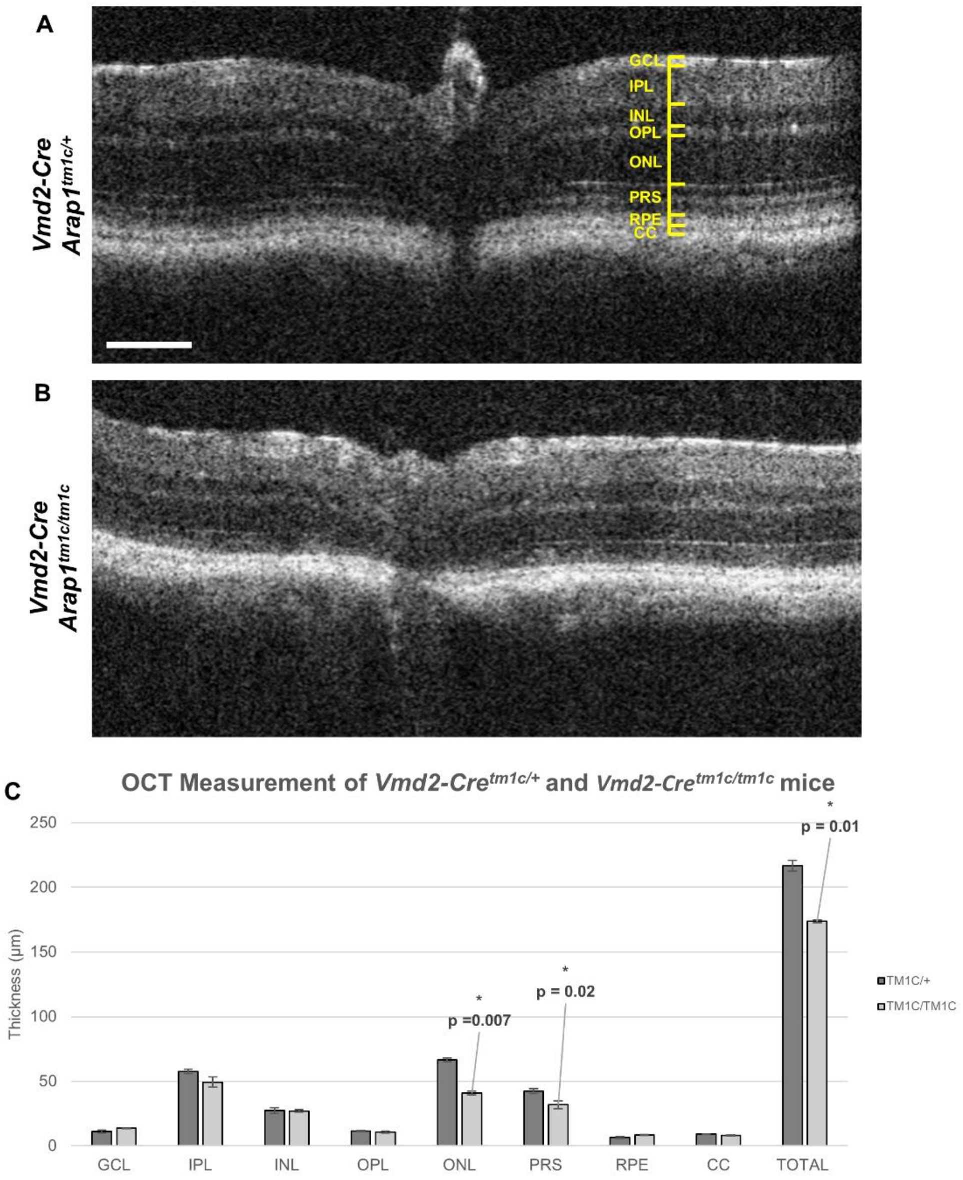

Fig. S2. **OCT quantification of Vmd2-Cre Arap1$^{tm1c/tm1c}$ and Vmd2-Cre Arap1$^{tm1c/+}$ mice***.* Vmd2-Cre Arap1$^{tm1c/tm1c}$ and Vmd2-Cre Arap1$^{tm1c/+}$ mice were analyzed with OCT at age 2.5 – 3 months. Example sections are shown for Vmd2-Cre Arap1$^{tm1c/+}$ (**A**) and Vmd2-Cre Arap1$^{tm1c/tm1c}$ mice (**B**). The ganglion cell layer (GCL), inner plexiform layer (IPL), inner nuclear layer (INL), outer plexiform layer (OPL), outer nuclear layer (ONL), inner segments (IS), photoreceptor layer (PRS), retinal pigment epithelium (RPE), and choriocapillaris (CC) are labeled (**A**). Retinal layers were measured and analyzed (**C**). Compared to Vmd2-Cre Arap1$^{tm1c/+}$ mice, Vmd2-Cre Arap1$^{tm1c/tm1c}$ mice demonstrated statistically significant thinning of the ONL and PRS layers, as well as a subsequent reduction in total retinal thickness (**C**). Scale bar (**A**) represents 100 µm. N = 3 for each group, tissue was collected from 3 different animals of each respective genotype, significance calculated by two-tailed Student's t-test, p values shown in graph, average values represent the mean, error bars represent s.e.m.

Disease Models & Mechanisms • Supplementary information

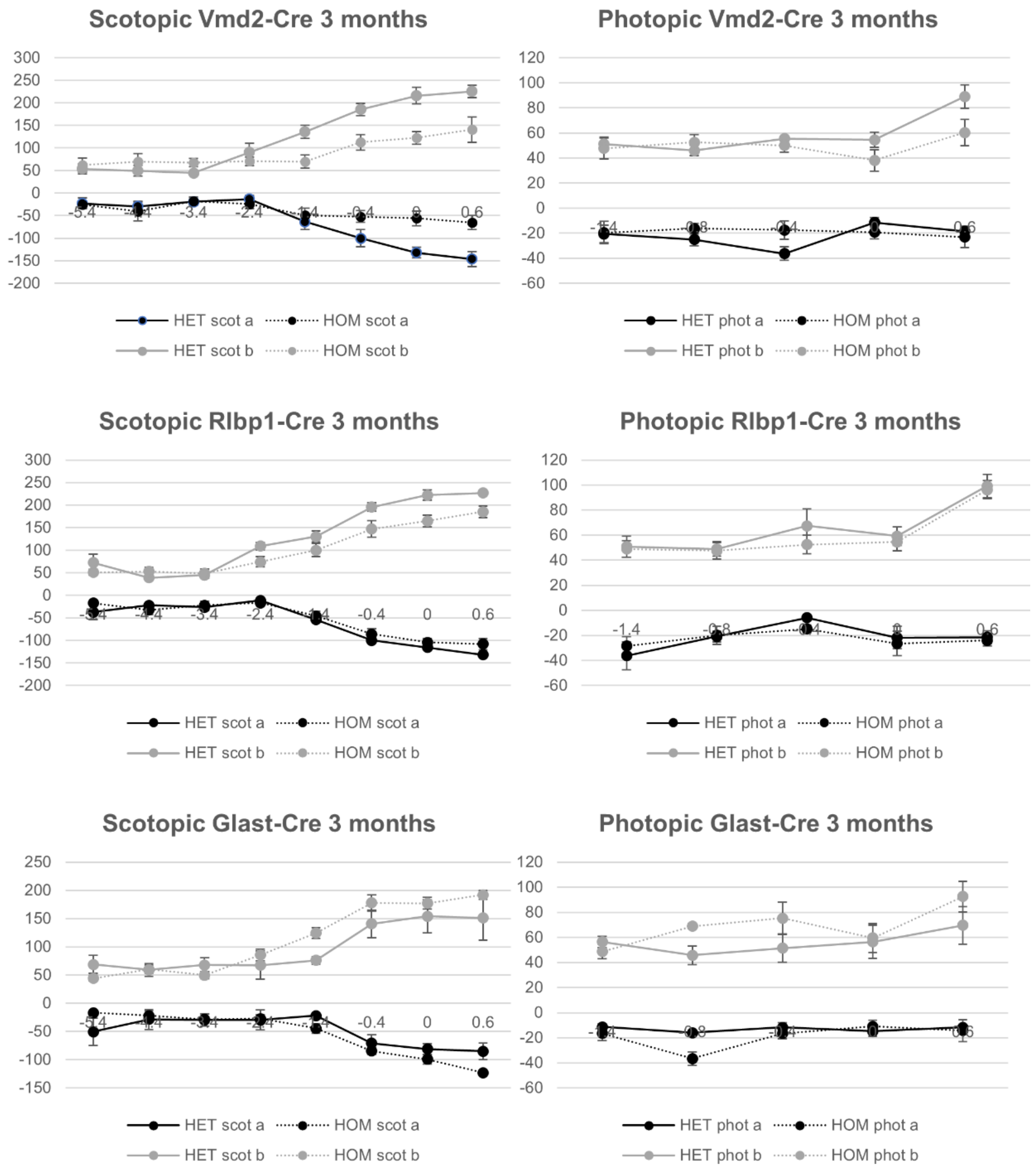

Fig. S3. **Full field electroretinography of Arap1 conditional knockout mice.** Vmd2-Cre tm1c/tm1c conditional KO mice (HOM, n=5) have reduced scotopic a-wave and b-wave magnitudes compared to Vmd2-Cre tm1c/+ controls (HET, n=5) indicative of reduced rod photoreceptor function. Reduced photopic b-wave magnitude is also seen, indicating some cone dysfunction as well (**A**). Glast-Cre tm1c/tm1c conditional KO mice (HOM, n=3) have comparable scotopic a-wave and b-wave magnitudes compared to Glast-Cre tm1c/+ controls (HET, n=3) indicative of normal rod and cone photoreceptor function (**B**). Rlbp1-Cre tm1c/tm1c conditional KO mice (HOM, n=8) have mildly reduced scotopic b-wave magnitudes compared to Rlbp1-Cre tm1c/+ controls (HET, n=5) indicative of reduced rod photoreceptor

Disease Models & Mechanisms • Supplementary information

function. Photopic magnitudes are normal, indicating no cone dysfunction at this stage (**C**). All animals are 3 months postnatal age. Vertical axes represent amplitude (µV). Horizontal axes represent stimulus intensity (log cd·s/m^2). Upper (gray) lines in each graph represent b-waves. Lower (black) lines in each graph represent a-waves. Error bars represent standard error of the mean. Tissues were collected from 5 different animals of each respective genotype in Vmd2-Cre analysis, 3 different animals of each respective genotype in Glast-Cre analysis, and 8 animals of each respective genotype in Rlbp1-Cre analysis.

Disease Models & Mechanisms • Supplementary information

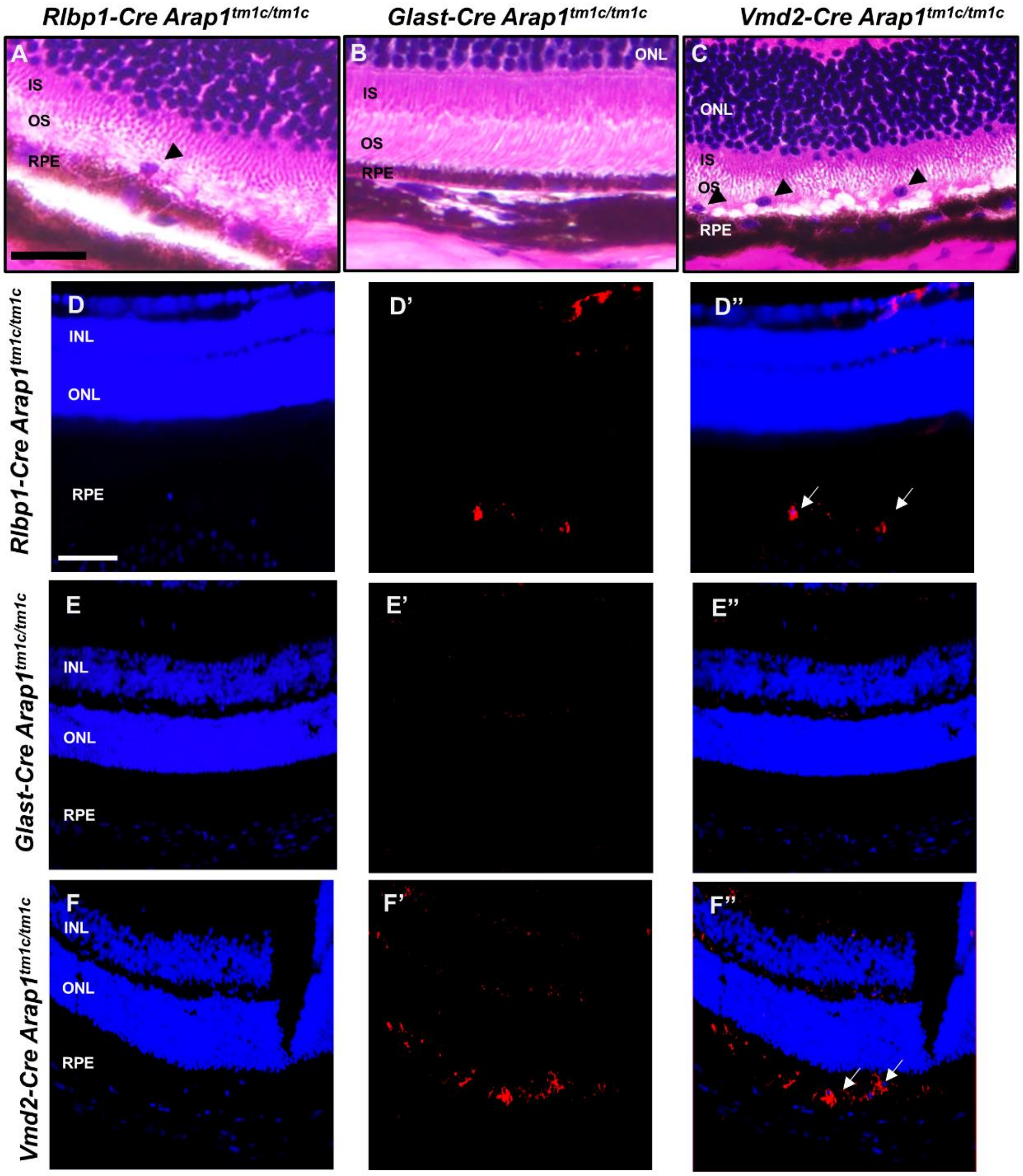

Fig. S4. Outer Retinal Macrophage Invasion in Cre cKO mice*.* Hematoxylin and eosin staining revealed cells suspicious for macrophages in the outer retina of *Vmd2-Cre Arap1*$^{tm1c/tm1c}$ and *Rlbp1-Cre Arap1*$^{tm1c/tm1c}$ mice (**A,C**; arrows), but absent in *Glast-Cre Arap1*$^{tm1c/tm1c}$ retinas (**B**). To confirm this finding, immunohistochemistry was performed with anti-CD11b (red) (**D',E',F'**) with DAPI (blue) (**D,E,F**) counterstaining to visualize the nuclei of the retinal layers in animals aged postnatal day 84. Channels were merged to create a composite image (**D'',E'',F''**). CD11b signal was detected in the outer retina of *Vmd2-Cre Arap1*$^{tm1c/tm1c}$ and *Rlbp1-Cre Arap1*$^{tm1c/tm1c}$ mice (**D'',F''**; arrows), indicative of macrophage invasion. *Glast-Cre Arap1*$^{tm1c/tm1c}$ retinas lacked any significant signal (**E''**). The inner nuclear layer (INL), outer nuclear layer (ONL), inner segments (IS), outer segments (OS), and retinal pigment epithelium (RPE) are labeled **(A, B, C, D, E, F)**. Images were taken at 40X magnification; scale bar represents 50 µm (A, B, C) and 100 µm **(D)**.

Disease Models & Mechanisms • Supplementary information

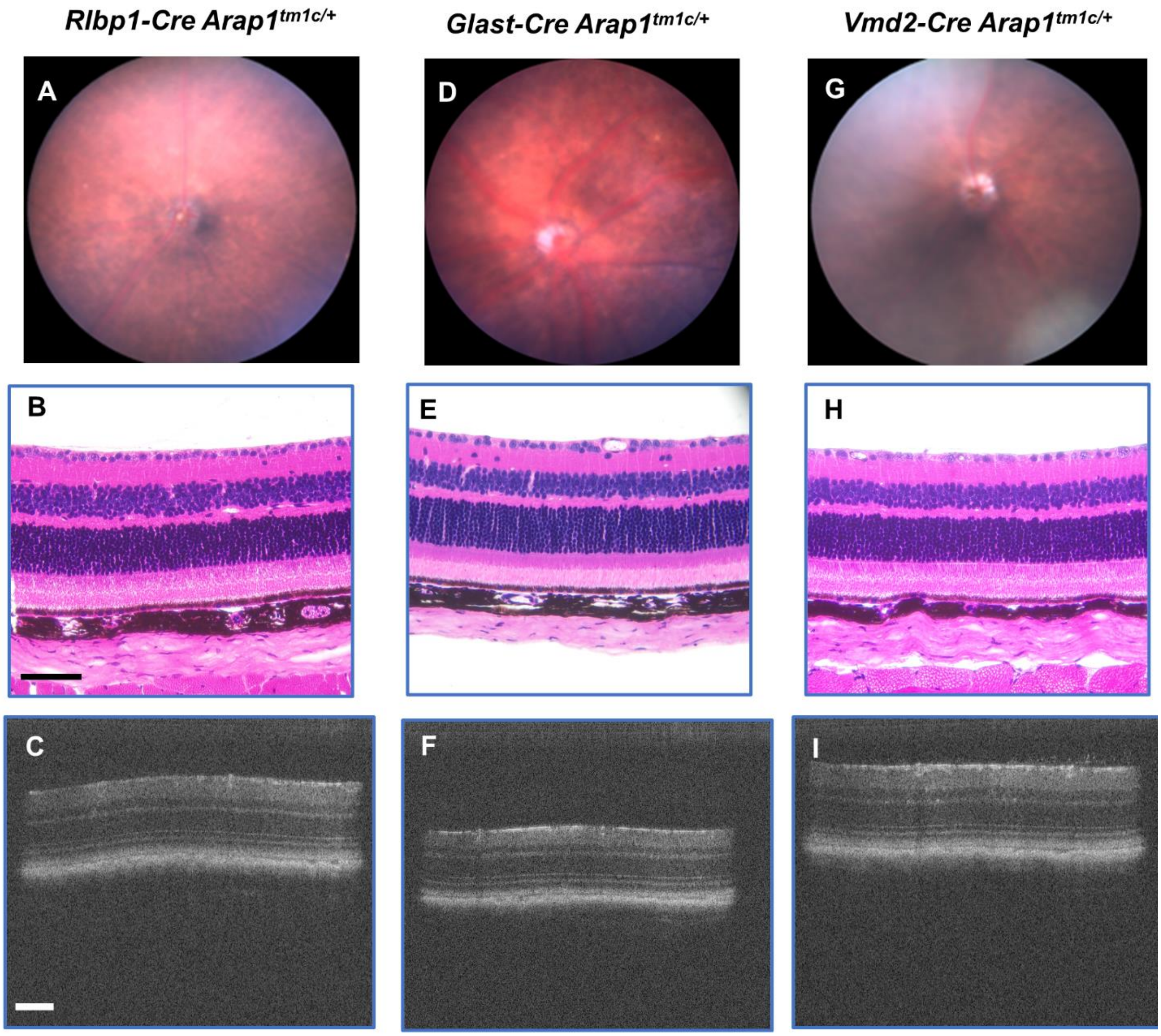

Fig. S5. Characterization of Cre$^{tm1c/+}$ mice. *Cre$^{tm1c/+}$* mice were analyzed with fundus photography, histology, and OCT analysis at 3 months of age (Glast-Cre, Rlbp1-Cre) and 1 month of age (Vmd2-Cre). Fundus photography, histopathology, and OCT analysis were all unremarkable. Quantification of retinal layers is shown in Figure 3C, 3G, and 3K.

Disease Models & Mechanisms • Supplementary information

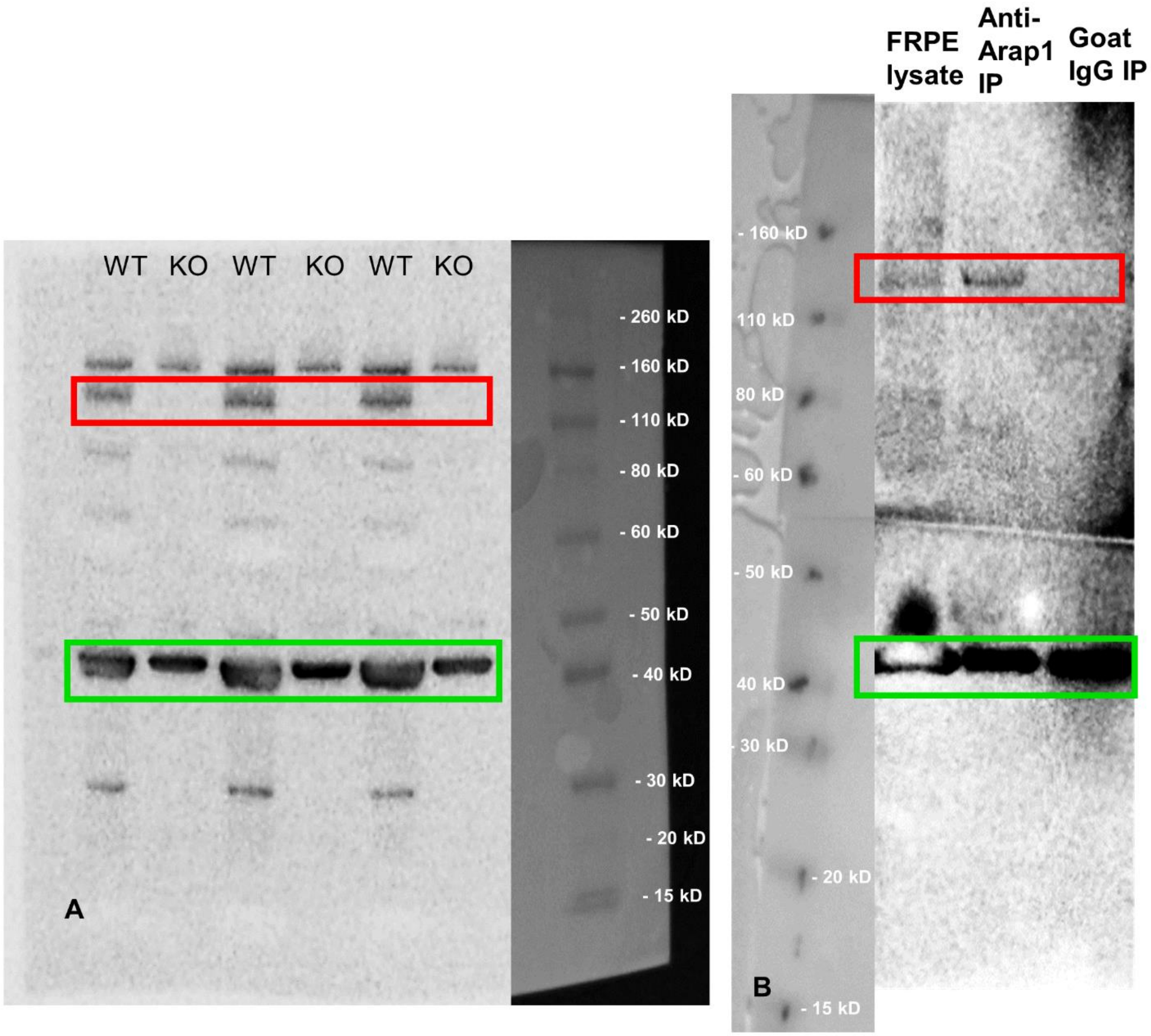

Fig. S6. Uncropped Blots. Immunoblot of WT and Arap1$^{-/-}$ mice in biological triplicate is shown (A) with Arap1 (Red) and β-actin (Green) outlined corresponding to the cropped blot in Figure 5A. Immunoblot of FRPE lysate, anti-ARAP1 immunoprecipitate from FRPE lysate, and goat IgG immunoprecipitate from FRPE lysate is shown (B) with Arap1 (Red) and β-actin (Green) outlined corresponding to the cropped blots in Figure 5B,C. Ladders are shown and labeled in both blots. Images of ladders were taken with monochromatic photography due to lack of colorimetric signal.

Disease Models & Mechanisms • Supplementary information

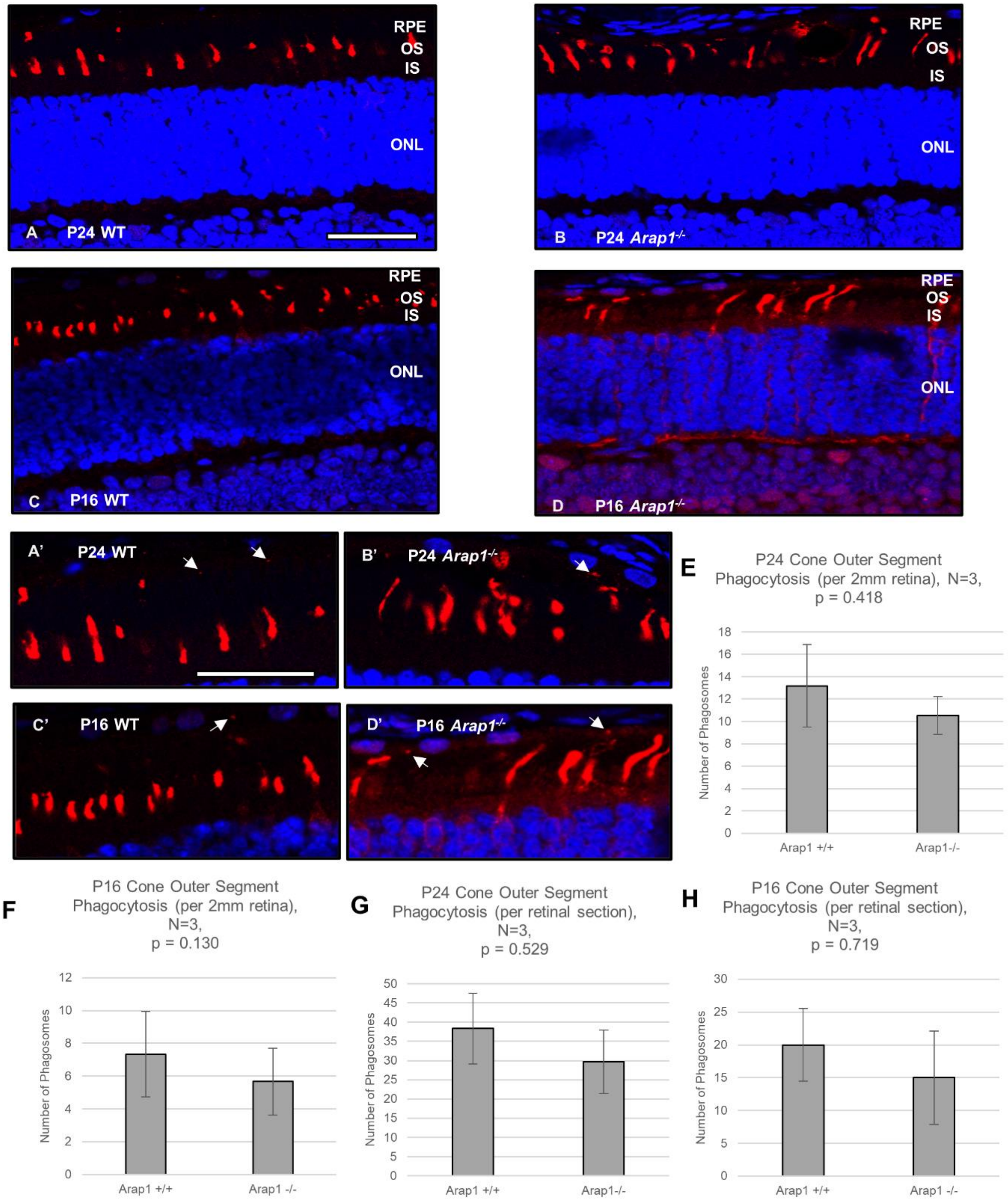

Fig. S7. **Quantification of RPE cone phagocytosis in Arap1$^{-/-}$ and WT mice**. Immunohistochemistry against anti-M and anti-L opsin (red) was used to quantify cone phagosomes. Sections were counterstained with DAPI to visualize the RPE nuclear layer (blue) of Arap1$^{-/-}$ and WT mice at P16 and P24 (A-D). Example cone phagosomes are shown (white arrowheads, A'-D'). Cone phagosomes were comparable between *Arap1$^{-/-}$* and wild type retinas

at both postnatal day 16 and 24 (**E-H**). Quantification of cone phagosomes is segregated into phagosomes per 2 mm retina (**E,F**) and absolute phagosome counts (**G,H**). Images were taken at 40x magnification; scale bar (white) represents 30 um. N = 3 each group, tissue collected from 3 different animals of each respective genotype, significance calculated by two-tailed Student's t-test, p values shown in graph, average values represent the mean, error bars represent s.e.m.

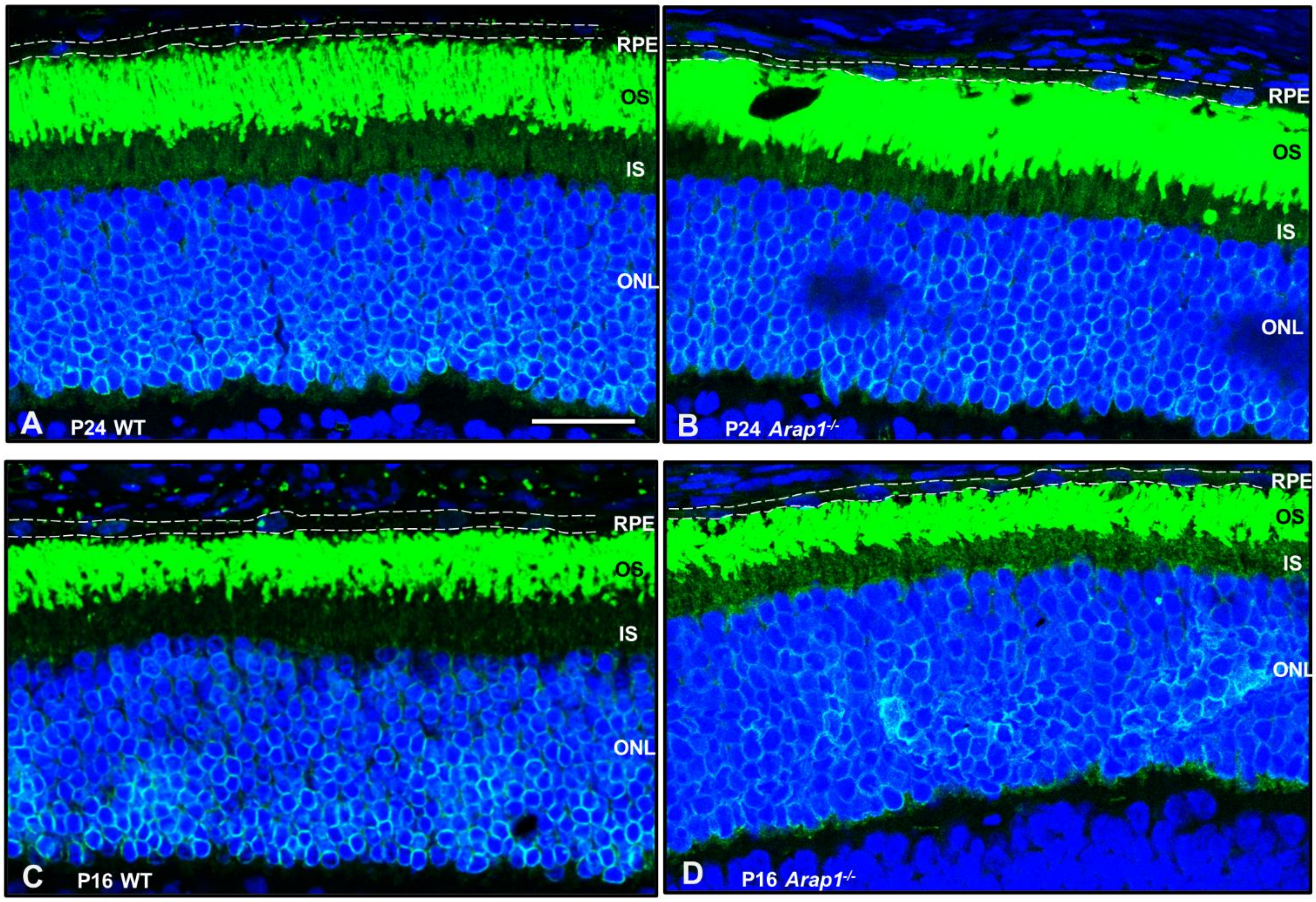

Fig. S8. Low Power Images of Rod Phagocytosis Quantification. Merged images of anti-rhodopsin immunosignal (green) and DAPI counterstaining (blue) are shown above corresponding to the images in Figure 6. The outer nuclear layer (ONL), photoreceptor inner segments (IS), photoreceptor outer segments (OS), and retinal pigment epithelium (RPE) layers are labeled. Boundaries of the RPE monolayer are shown in each section (white hashed lines). Images were taken at 40x magnification; scale bar (white) represents 30 um.

Disease Models & Mechanisms • Supplementary information

Table S1. Summary of primary antibodies

Antibody	Catalogue Number	Dilution	PMID
Goat anti-Arap1	ab5912	1:50 IP, 1:2000 WB	19589776, 30240610
Normal goat IgG	AB-108-C	1:50 IP	28697340, 29195074, 29712778, 30105032, 30170813, 30290143, 30293776, 30590048, 30605666, 30661803, 31050726, 31085177, 31189102, 31242418, 31273787, 31543445, 31746735, 31813798, 31956038, 32579116
Rabbit anti-Beta actin	4970S	1:4000 WB	23671263, 25514086, 26295370, 26572741, 26672806, 27003841, 27100620, 27320125, 27566322, 27641501, 27863209, 27935476, 28111073,

Disease Models & Mechanisms • Supplementary information

			28193684,<br>28286049,<br>28318489,<br>28323956,<br>28388439,<br>28407483,<br>28434841,<br>28467927,<br>28520923,<br>28709002,<br>28768176,<br>28803777,<br>28867293,<br>28919040,<br>28978427,<br>29136504,<br>29229708,<br>29307841,<br>29331024,<br>29364516,<br>29429824,<br>29533785,<br>29546371,<br>29551594,<br>29576452,<br>29576536,<br>29594258,<br>29703257,<br>29727620,<br>29763624,<br>29796592,<br>29858554,<br>29894691,<br>29958804,

Disease Models & Mechanisms • Supplementary information

			29990501, 30017355, 30100196, 30119679, 30146486, 30150364, 30157429, 30170813, 30197082, 30224541, 30230471, 30269950, 30299260, 30300582, 30304388, 30318461, 30401435, 30416750, 30458137, 30463012, 30463018, 30537512, 30578080, 30661757, 30686770, 30738829, 30745181, 30773463, 30827895, 30851189, 30930167, 30943409, 30951171, 30970261,

Disease Models & Mechanisms • Supplementary information

			31031094, 31067464, 31077342, 31113010, 31135337, 31287417, 31339573, 31504388, 31509750, 31509751, 31581797, 31593505, 31606272, 31663064, 31665623, 31708446, 31722204, 31734944, 31747595, 31775025, 31775054, 31794717, 31801089, 31809714, 31859249, 31918402, 31954709, 32037591, 32053707, 32127496, 32154743, 32251678, 32376600, 32396064, 32398749, 32851972

Disease Models & Mechanisms • Supplementary information

mouse anti-rhodopsin	MABN15	1:1000 IHC	28345128, 31067458, 31139088
rabbit anti-L/M opsin	AB5405	1:1000 IHC	17183536, 17990268, 18072193, 18076080, 18425804, 20029995, 20593360, 21246551, 21681749, 24737644, 24920619, 27020758, 28324012, 28542922, 28815501, 28839191, 29106373, 29174891, 29456141, 30332642, 30560780, 31091454, 31438467, 32123325, 32463363

Catalogue numbers, dilutions, and PMID’s of previously published papers using each respective antibody are provided. IP = immunoprecipitation, WB = western blot, IHC = immunohistochemistry.

Disease Models & Mechanisms • Supplementary information

Table S2. Notable proteins from LC-MS/MS analysis.

**Name**	**Symbol**	**Protein Molecular Weight (kDa)**	**Unique Peptide Count**	**Percentage Sequence Coverage (%)**
SH3 domain-containing kinase-binding protein 1	CIN85	73.1	1	2
Cell division control protein 42	CDC42	21.2	1	11
Ras-related C3 botulinum toxin substrate 1	RAC1	21.4	2	18

The proteins above are proteins with notable interactions with ARAP1 corroborated by literature search but were unable to meet inclusion criteria. Protein name, symbol, molecular weight are provided, as well as number of unique peptides and percentage of sequence coverage detected by mass spectrometry.
